# Circadian regulator REV-ERBα is a master regulator of tumor lineage plasticity and an effective therapeutic target

**DOI:** 10.1073/pnas.2513468122

**Published:** 2025-11-13

**Authors:** Xiong Zhang, Yatian Yang, Hongye Zou, Demin Cai, Eva Corey, Amina Zoubeidi, Su Hao Lo, Ai-Ming Yu, Ronald M. Evans, Hong-Wu Chen

**Affiliations:** ^a^Department of Biochemistry and Molecular Medicine, School of Medicine, University of California Davis, Davis, CA 95817; ^b^Department of Urology, University of Washington, Seattle, WA 98195; ^c^Department of Urologic Sciences, Faculty of Medicine, University of British Columbia, Vancouver, BC V6H 3Z6, Canada; ^d^Gene Expression Laboratory, Salk Institute for Biological Studies, La Jolla, CA 92037; ^e^Comprehensive Cancer Center, University of California Davis, Sacramento, CA 95817; ^f^Veterans Affairs Northern California Health Care System-Mather, Mather, CA 95655

**Keywords:** REV-ERBα, NEPC, lineage plasticity, drug resistance, therapeutic targeting

## Abstract

Epigenetic and transcriptional dysregulation plays a fundamental role in tumor lineage plasticity (LP). However, the underlying mechanisms, especially for the initial events of LP development, are still poorly understood. Here, we report that in progression of prostate cancer from adenocarcinoma to treatment-induced neuroendocrine prostate cancer (t-NEPC), anti-androgen receptor (AR) signaling inhibitors (ARSIs) reprogram the function of circadian regulator/nuclear receptor REV-ERBα by switching its target gene programs from kinase signaling and metabolic programs to programs of LP, which includes neurogenesis, stem cell, and epithelial–mesenchymal transition as well as over fifteen LP drivers including POU3F2/BRN2, ASCL1, FOXA2, ONECUT2, and MYCN. Unexpectedly, REV-ERBα facilitates the chromatin occupancy of BRN2, ASCL1, and FOXA1 in their activation of LP programs, thus functioning as a master regulator of ARSI-induced LP driver network. Mechanistically, REV-ERBα induces chromatin accessibility and H3K27ac modification at promoters of LP genes through its recruitment of BRD4 and p300. Overexpression of REV-ERBα alone is sufficient to induce LP and neuroendocrine phenotype and confers resistance to ARSI in adenocarcinoma cells. Loss of REV-ERBα potently inhibits NEPC cell growth and abolishes the expression of LP drivers and gene programs. Pharmacological inhibition of REV-ERBα exhibits high potency in blocking the growth of NEPC tumors including patient-derived xenografts. Our findings reveal that therapy-induced LP development entails a coordinated induction of a network of LP drivers and that REV-ERBα is an unexpected master regulator of the network and a promising therapeutic target for treatment of advanced prostate cancer such as NEPC.

## Significance

Tumor lineage plasticity (LP) is a major mechanism of therapy resistance and metastasis in many solid tumor malignancies including prostate cancer. Better understanding of the process is vital to developing effective therapeutic intervention. Here, we report that drugs targeting the androgen receptor (AR) in prostate cancer induce a functional switch of circadian regulator/nuclear receptor REV-ERBα to act as a master regulator in the initial induction of a network of LP driving factors. Pharmacological or genetic inhibition of REV-ERBα potently blocks the growth of drug-resistant tumors and effectively suppresses tumor LP, thus nominating REV-ERBα as an attractive target for treatment of advanced diseases with LP.

Prostate cancer (PCa) tumor growth and progression are largely dependent on deregulated androgen receptor (AR) function at initial diagnosis (1–3). Thus, androgen deprivation therapy (ADT) and anti-AR signaling inhibitors (ARSIs) such as enzalutamide (ENZ) are widely used therapeutic options (4, 5). However, most treated patients will have their disease progressed to metastatic castration-resistant prostate cancer (mCRPC) (6–8). Tumors relapsed often display diverse cellular and molecular phenotypes in the form of lineage plasticity (LP) where a subpopulation of tumor cells express one or more of nonluminal epithelial lineage gene programs including those of neuroendocrine that gives rise to treatment-induced neuroendocrine PCa or treatment-induced neuroendocrine prostate cancer (t-NEPC), neuronal, stem-like, or mesenchymal lineages (9–11). Most of the treatment-relapsed tumors display low or altered AR function while some become double-negative (negative for both AR and NE feature) PCa (DNPC) (12). With the wide use of highly potent ARSI at the clinic, the incidence of t-NEPC and tumors displaying marked LP appears ever increasing (13–17). However, no effective therapy is available for the deadly progressed disease (18, 19).

While genetic alterations, such as loss of function of tumor-suppressor genes PTEN, TP53, and RB1, may make tumor cells permissible for LP in general, nongenetic mechanisms such as epigenetic or transcriptional dysregulation have been increasingly recognized as the major contributors (20–23). Indeed, several transcription factors (TFs), particularly those that play crucial roles in developmental neurogenesis (e.g., N-myc proto-oncogene protein (MYCN), forkhead box protein A2 (FOXA2), POU domain, class 3, transcription factor 2 (POU3F1/BRN2), achaete-scute family bHLH transcription factor 1 (ASCL1), neuronal differentiation 1 (NEUROD1), SRY-box transcription factor 2 (SOX2), and one cut homeobox 2 (ONECUT2)) have been shown to drive LP in preclinical models (24–32). However, readily druggable targets that drive LP are still urgently needed. Moreover, the questions of how those LP-driving TFs are induced and functionally regulated are also poorly understood.

Reverse c-erbA alpha (REV-ERBα), encoded by nuclear receptor subfamily 1 group D member 1 (NR1D1) gene, is a TF that plays important roles in a range of physiological processes including regulation of energy metabolism, development, immunity as well as circadian rhythm (33–38). Our previous study found that REV-ERBα switches its physiological function from a repressor to a master activator in adenocarcinoma of PCa and liver cancer to directly control multiple tumorigenic programs including MAPK and PI3K-Akt signaling, through partnering with BRD4/p300 coactivators and FOXA1 (39).

In this study, we identify REV-ERBα as a master regulator in the initiation and propagation of LP programs in PCa cells and tumors. We found that ARSI reprograms its function from controlling kinase signaling programs in adenocarcinoma to directly stimulating the expression of more than 10 major LP drivers and the LP programs. Remarkably, REV-ERBα facilitates the function of other drivers such as BRN2 and ASCL1, thus serving as a master regulator of ARSI-induced LP driver network. Loss of REV-ERBα or its pharmacological inhibition potently blocks NEPC tumor growth and abolishes tumor LP feature, thus making REV-ERBα an attractive therapeutic target for effective treatment of advanced tumors featuring LP such as NEPC.

## Results

### Elevated Expression of AR-Suppressed NR1D1/REV-ERBα Is Associated with Tumor LP.

**Fig. 1.**
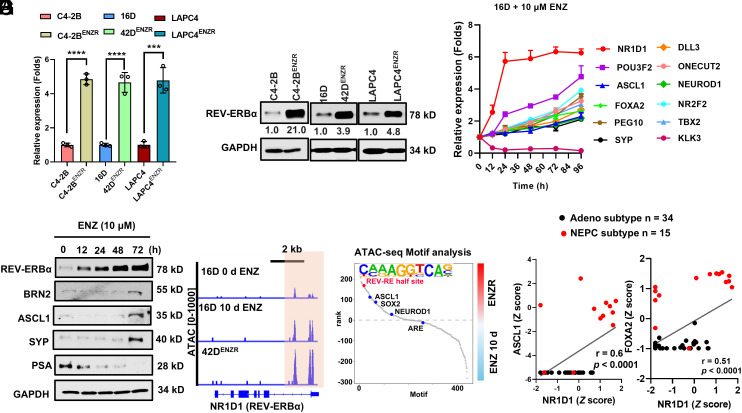
Elevated expression of AR-suppressed NR1D1/REV-ERBα is associated with tumor LP. (*A* and *B*) q-RT PCR (*A*) and immune blotting analysis (*B*) of expression of NR1D1 mRNA and protein in ENZR cells and their parental cells. The intensity of REV-ERBα bands was normalized to GAPDH. The ENZR cells were maintained in the presence of 10 μM ENZ. (*C* and *D*) q-RT PCR (*C*) and immunoblotting (*D*) analysis of NR1D1 and LP drivers POU3F2/BRN2, ASCL1, FOXA2, PEG10, DLL3, ONECUT2, NEUROD1, NR2F2/COUP-TF2, and TBX2 and neuroendocrine marker SYP in CRPC 16D cells treated with 10 μM ENZ for indicated hours. AR target gene KLK3 and its encoded protein PSA was used as an indicator of AR activity. (*E*) IGV snapshot displays changes of chromatin accessibility at NR1D1 chromatin regions in 16D cells treated with 10 μM ENZ for 10 d and its ENZR subline 42D^ENZR^ cells. Data are from GSE183200. (*F*) TF binding motifs surrounding accessible chromatin in 42D^ENZR^ vs. 16D cells treated with 10 μM ENZ for 10 d, ranked based on differential *P*-value. Data are from GSE183200. (*G*) Correlations between the expression of NR1D1 and LP drivers in the 2016 Beltran cohort.

Previously, we found that REV-ERBα switches its functions from a transcriptional repressor to a protumorigenic activator in prostate adenocarcinoma (39). In our continued studies of REV-ERBα in PCa, we observed that the messenger RNA (mRNA) and protein expression of NR1D1/REV-ERBα is markedly upregulated in three different ARSI enzalutamide (ENZ)-resistant (ENZR) cells (Fig. 1 *A* and *B*). Further examination revealed that ENZ treatment induced the expression of NR1D1/REV-ERBα and LP drivers, such as BRN2, ASCL1, FOXA2, and ONECUT2, in a dose and time-dependent manner in adenocarcinoma cells, with REV-ERBα being the fastest and strongest induced among them (Fig. 1 *C* and *D* and *SI Appendix*, Fig. S1 *A*–*C*). Interestingly, like POU3F2/BRN2 being an AR-repressed gene (25), ENZ treatment diminished AR binding at the downstream enhancer of NR1D1 and increased gene-activating H3K27ac mark at both its enhancer and promoter (*SI Appendix*, Fig. S1 *G* and *H*). Consistently, ENZ treatment or resistance to ENZ is associated with an increased chromatin accessibility at the NR1D1 and LP marker CHGA promoter regions (Fig. 1*E* and *SI Appendix*, Fig. S1*I*). To determine whether REV-ERBα is directly regulated by androgen signaling, we treated AR-positive prostate cancer cells (16D and C4-2B) with increasing concentrations of dihydrotestosterone (DHT). DHT treatment led to a dose-dependent suppression of REV-ERBα expression, accompanied by an increase in AR and the canonical AR target gene KLK3/PSA in both models (*SI Appendix*, Fig. S1 *D* and *E*). Indeed, NR1D1 expression is also elevated in clinical tumors treated by abiraterone and enzalutamide (*SI Appendix*, Fig. S1*F*) (40–42). These findings indicate that AR activation negatively regulates REV-ERBα, suggesting a feedback mechanism in which androgen signaling counteracts REV-ERBα induction and thereby modulates tumor LP programs. TF motif analysis of ATAC-seq peaks induced by ENZ treatment or in NEPC cells revealed that REV-RE-like, half-site motif (CA/CA/GAGGTCA) that we previously identified in prostate cancer (39) was enriched in hyperaccessible chromatin regions in ENZR cells and post–ENZ treatment (Fig. 1*F* and *SI Appendix*, Fig. S1*J*). Significantly, NR1D1 is elevated in NEPC tumors when compared with adenocarcinoma (*SI Appendix*, Fig. S1*K*). Its expression is significantly correlated with the expression of LP drivers, such as ASCL1, FOXA2, NR2F1(COUP-TF1), SOX2, PEG10, and NE markers CHGA (Fig. 1*G* and *SI Appendix*, Fig. S1*L*). These data suggest that NR1D1/REV-ERBα is an unexpected AR-suppressed gene and that its elevated expression is associated with tumor LP.

### REV-ERBα Plays an Essential Role in ARSI Induction of a LP Driver Network and LP Programs.

**Fig. 2.**
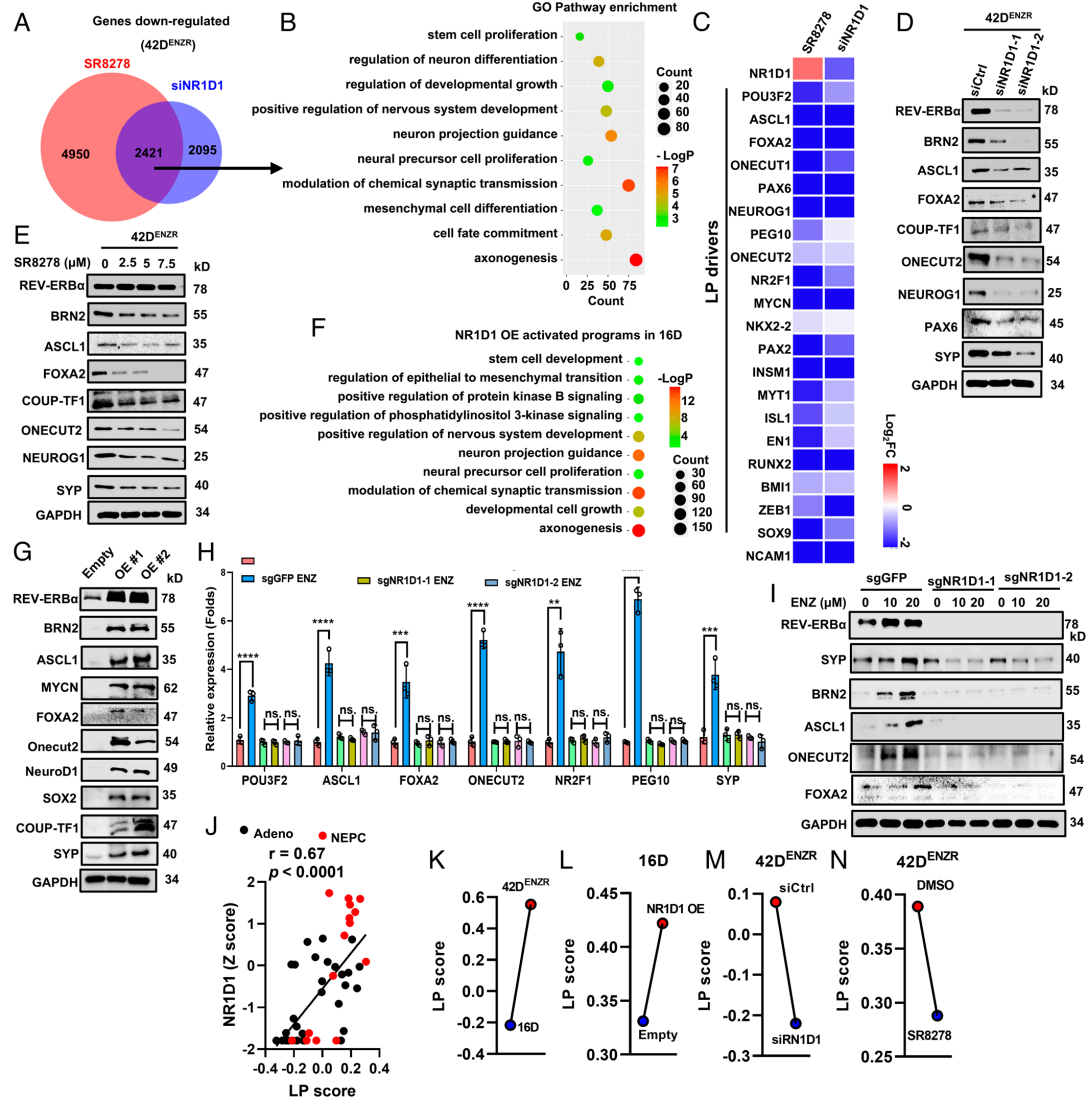
REV-ERBα plays an essential role in ARSI induction of LP gene programs. (*A*) Venn diagram of the number of genes with expression significantly (>1.5-fold) downregulated in 42D^ENZR^ cells treated with siRNA against NR1D1 and 7.5 μM antagonist SR8278 for 48 h. (*B*) Gene ontology (GO) analysis of the commonly downregulated genes by siRNA knockdown and antagonist treatment. Top 10 representative programs were shown. (*C*) Heatmap shows mRNA expression changes of LP drivers. (*D* and *E*) Immunoblotting analysis of REV-ERBα and LP drivers and marker in 42D^ENZR^ cells treated with REV-ERBα siRNA (*D*) and 7.5 μM antagonist SR8278 (*E*) for 48 h. (*F*) GO analysis of the upregulated genes (>1.5-fold) in NR1D1 ectopic OE cells. Top 10 representative programs were shown. (*G*) Immunoblotting analysis of indicated proteins in 16D CRPC cells with NR1D1/REV-ERBα OE. (*H* and *I*) q-RT PCR (*H*) and Immunoblotting analysis (*I*) of indicated genes and proteins in response to 10 μM ARSI ENZ treatment for 4 d in C4-2B cells with NR1D1/REV-ERBα KO. Student’s *t* test. **P < 0.01, ***P < 0.001, ****P < 0.0001. (*J*) Correlations between the expression of NR1D1 and LP signature score generated by GSVA. (*K*–*N*) Dot plots showing changes of LP signature scores for 16D and 42D^ENZR^ (*K*), 16D cells with NR1D1 OE (*L*), 42D^ENZR^ with NR1D1 KD (*M*), and 42D^ENZR^ treated with 7.5 μM antagonist SR8278 (*N*).

To examine how REV-ERBα controls tumorigenic gene programs, we performed RNA-seq analysis of 42D^ENZR^ cells treated with siRNA against NR1D1/REV-ERBα or REV-ERBα antagonist SR8278 (39). 42D^ENZR^ cells are considered to be treatment-induced NEPC (t-NEPC) model as they express a number of LP drivers and markers (43). Analysis of genes with expression significantly down-regulated by the two treatments showed a high concordance with around 50% of genes altered by both treatments (Fig. 2*A*). GO analysis of the commonly down-regulated transcripts revealed that genes involved in LP gene programs of neurogenesis, axon guidance, developmental cell growth, stem cell proliferation, and epithelial–mesenchymal transition (EMT) were among the most highly enriched (Fig. 2*B* and Dataset S1). Consistent with our previous findings (39), REV-ERBα antagonist SR8278 and REV-ERBα knockdown (KD) up-regulated the expression of genes enriched in programs of steroid metabolic process, immune response, and p53 signaling (*SI Appendix*, Fig. S2 *A* and *B* and Dataset S1). Our pathway-focused analysis demonstrated that a major proportion of genes in the LP programs including axonogenesis (84 genes out of 460 in the program), neuron projection guidance (54 genes out of 278), stem cell proliferation (16 genes out of 64), and EMT (37 genes out of 231) were significantly down-regulated by REV-ERBα KD and the REV-ERBα antagonist (Fig. 2*B* and Dataset S1). Notably, they include more than 15 established and putative LP drivers (e.g., POU3F2/BRN2, ASCL1, FOXA2, ONECUT1/2, MYCN, NEUROG1, NEUROD1, NKX2-2, NR2F1/COUP-TFI, ZEB1, RUNX2, EN1, PAX2/6, SOX9, and BMI1) (Fig. 2*C*). Consistently, REV-ERBα KD and its antagonist treatment resulted in a strong downregulation of protein expression of LP drivers (e.g., BRN2, ASCL1, FOXA2, ONECUT2, COUP-TF1, and NEUROG1) and marker (SYP) (Fig. 2 *D* and *E* and *SI Appendix*, Fig. S2*C*).

Our previous study revealed that REV-ERBα inverts its function from a repressor to directly activate over 3,000 tumorigenic genes involved in MAPK and PI3K-Akt signaling, lipid metabolism, and cell cycle programs (39). Interestingly, except for cell cycle/ DNA replication programs, the other major REV-ERBα-activated programs, such as the kinase signaling and lipid metabolism are not significant enriched in genes downregulated by REV-ERBα KD in the 42D^ENZR^ cells (*SI Appendix*, Fig. S2*D*). Together with the data described above, our results demonstrated that REV-ERBα function is reprogrammed in prostate cancer progression from activating tumorigenic kinase signaling and metabolic programs in adenocarcinoma to activating LP gene programs in NEPC.

To further characterize the function of REV-ERBα, we ectopically expressed REV-ERBα in ARSI-sensitive 16D cells (25). Overexpression (OE) of REV-ERBα strongly stimulated the expression of LP gene programs including stem cell development, EMT, nervous system development, and axonogenesis, as well as LP drivers BRN2, ASCL1, MYCN, FOXA2, ONECUT2, NEUROD1, SOX2, and COUP-TF2 and markers (e.g., SYP and CHGA) (Fig. 2 *F* and *G*, *SI Appendix*, Fig. S2*E*, and Dataset S1). To further validate the role of REV-ERBα in regulating LP drivers, we performed qRT-PCR analyses in multiple perturbation models. REV-ERBα OE led to a marked upregulation of those LP-associated genes (*SI Appendix*, Fig. S2*F*). Conversely, pharmacological inhibition of REV-ERBα with its antagonist SR8278, as well as siRNA-mediated KD, significantly reduced the expression of core LP drivers including ASCL1, BRN2, ONECUT2, NEUROD1, and FOXA2 and LP markers SYP and NCAM1, consistent with our RNA-seq results (*SI Appendix*, Fig. S2 *G* and *H*). Together, these findings establish that REV-ERBα is both necessary and sufficient to activate a network of LP drivers, and that pharmacological blockade is sufficient to suppress their expression. To determine whether REV-ERBα is a master regulator for initiation of LP, we generated NR1D1/REV-ERBα KO cells using CRISPR-Cas9 system and treated the cells with ARSI enzalutamide (ENZ), Apalutamide (Apal), Bicalutamide (Bica), and Darolutamide (Daro). As shown in Fig. 2 *H* and *I* and *SI Appendix*, Fig. S2 *I* and *J*, KO of NR1D1 effectively blocked the ARSIs induction of LP drivers and markers. To further evaluate LP, we applied Gene Set Variation Analysis (GSVA) using the GOBP_NEURON_DIFFERENTIATION gene set from MSigDB, which encompasses key LP drivers and markers including POU3F2/BRN2, ASCL1, NEUROD1, NEUROG1, and FOXA2. Analysis of the Beltran dataset (17) revealed a significant positive correlation between NR1D1/REV-ERBα expression and LP signature scores (Fig. 2*J*). Consistently, LP signature score was markedly elevated in 42D^ENZR^ cells compared with their parental counterparts (Fig. 2*K*). Moreover, REV-ERBα OE further increased LP signature scores (Fig. 2*L*), whereas both genetic knockdown of NR1D1/REV-ERBα and pharmacological inhibition with the REV-ERBα antagonist SR8278 significantly reduced LP scores (Fig. 2 *M* and *N*), supporting a functional role for NR1D1/REV-ERBα in driving ARSI-induced LP. Next, we examined whether REV-ERBα is reciprocally regulated by the LP drivers. Interestingly, as shown in *SI Appendix*, Fig. S2*K*, KD of FOXA1, ASCL1, and BRN2 had no effects on the expression of REV-ERBα, indicating REV-ERBα is an upstream TF for induction of the other LP drivers. Together, the results demonstrated that REV-ERBα plays an essential role in ARSI induction of LP gene programs including its stimulation of a network of LP drivers.

### Elevated REV-ERBα Promotes ARSI Resistance and Aggressive Cellular Features That Are Associated with Tumor LP.

**Fig. 3.**
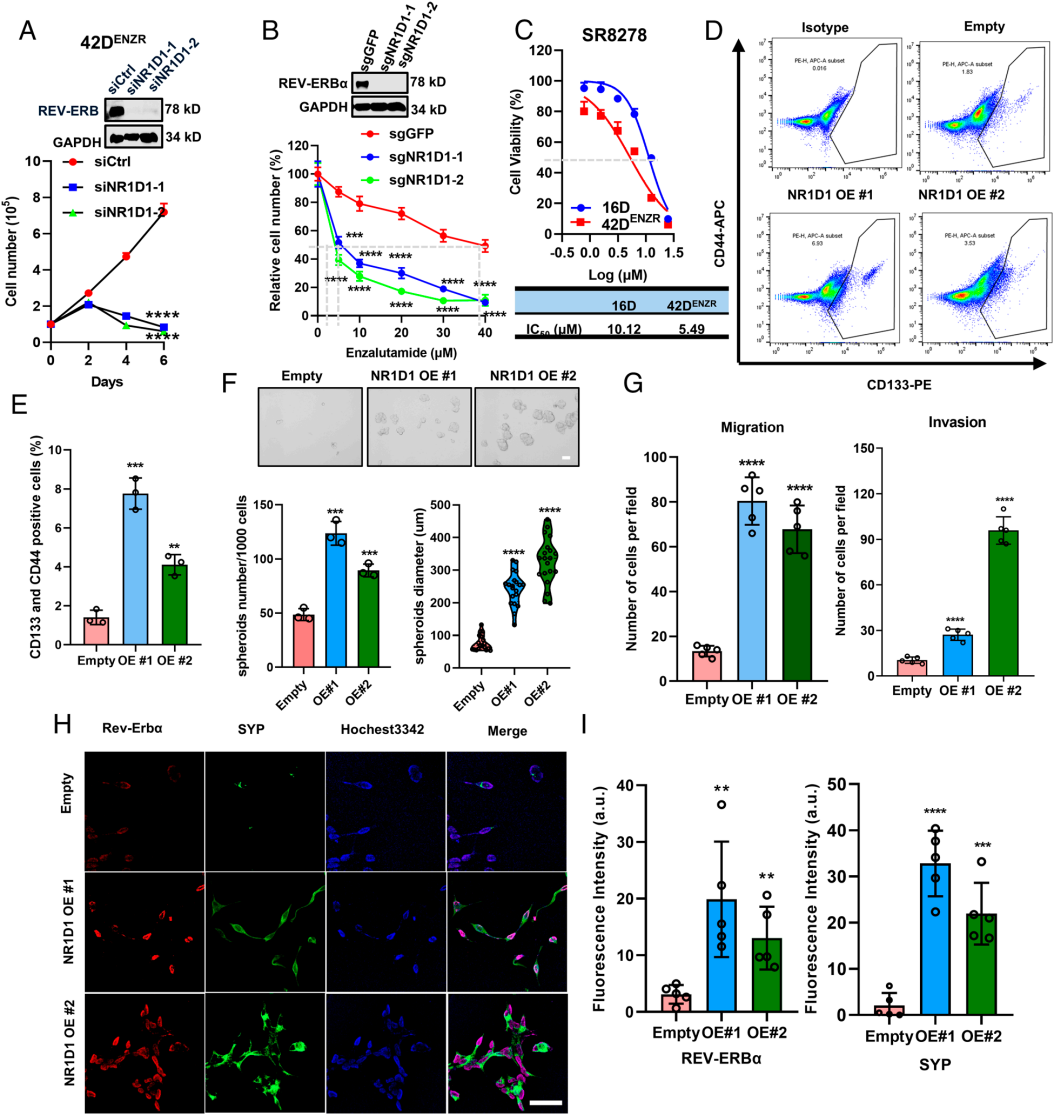
Elevated REV-ERBα promotes ARSI resistance and aggressive cellular features that are associated with tumor LP. (*A*) Viable cell numbers were measured for t-NEPC 42D^ENZR^ cells transfected with indicated siRNAs. (*B*) Viable cell numbers were measured for C4-2B cells with NR1D1 gene knockout or control cells treated with indicated concentrations of ENZ for 4 d. KO efficacy of NR1D1 gene was measured using immunoblotting. (*C*) Cell-Titer Glo assays of cell viability of 42D^ENZR^ and its parental CRPC16D cells treated with indicated concentrations of REV-ERBα antagonist SR8278 for 4 d. IC_50_ values of 42D^ENZR^ and its parental CRPC16D cells in response to SR8278 were calculated using GraphPad Prism software. (*D* and *E*) Representative flow cytometry plots of stem cell population (CD44pos and CD133pos) in 16D cells with REV-ERBα OE (*D*). Plot shows the quantification of stem cell populations (*E*). (*F*) Representative images of in vitro tumor sphere formation in 16D cells with REV-ERBα OE. Sphere number was counted and sphere diameter was measured using Image J software. (Scale bar, 200 μm.) (*G*) Migrated and invaded cells were stained and visualized by phase-contrast microscopy (10× magnification). Numbers of migrated and invaded cells were counted. (*H* and *I*) Fluorescence microscope images of REV-ERBα (red) and SYP (green) in 16D cells with REV-ERBα OE. The staining intensity was measured using Image J software (*I*). (Scale bar, 50 μm.) In panels *A*, *B*, *E*, *F*, *G*, and *I*, Student’s *t* test was performed. **P < 0.01, ***P < 0.001, ****P < 0.0001.

Next, we examined the function of elevated REV-ERBα in ARSI resistance. KD of REV-ERBα significantly reduced the cell growth of ARSI-resistant 42D^ENZR^, C4-2B^ENZR^, and LAPC4^ENZR^ cells (Fig. 3*A* and *SI Appendix*, Fig. S3*A*). NR1D1 CRISPR KO significantly sensitized adenocarcinoma cells to ARSI ENZ with IC_50_ reduced from 30 µM to approximately 5 µM (Fig. 3*B*). We also treated cells with REV-ERBα antagonist SR8278 and found that SR8278 can potently inhibit the growth of ARSI-resistant cells. Intriguingly, the resistant cells displayed higher sensitivity to the antagonist than the parental cells based on the IC_50_ measurements (Fig. 3*C* and *SI Appendix*, Fig. S3 *B* and *C*). Moreover, SR8278 also potently inhibited the growth of cells derived from a de novo NEPC PDX tumor (*SI Appendix*, Fig. S3*D*).

To further examine the function of REV-ERBα in ARSI resistance, we ectopically expressed REV-ERBα in three different ARSI-sensitive adenocarcinoma cells (16D, C4-2, and LAPC4). REV-ERBα OE strongly promoted ARSI resistance in the three cell models. A high concentration of 40 µM ENZ reduced the cell number to about 10% in the control cells but to less than 50% in the OE cells (*SI Appendix*, Fig. S3*E*). Moreover, REV-ERBα OE significantly promoted resistance to multiple ARSIs, as reflected by sustained cell viability in the presence of other ARSIs Apalutamide, Bicalutamide, and Darolutamide (*SI Appendix*, Fig. S3*F*), indicating NR1D1/REV-ERBα confers broad resistance to AR-targeted therapies. Consistent with its function in activating LP programs, REV-ERBα OE significantly increased stem-like cell population and spheroid formation. Its OE also significantly enhanced cell migration and invasion activities. In contrast, REV-ERBα KD or its antagonist significantly reduced the stem-like cell population and spheroid formation as well as cell migration and invasion activities (Fig. 3 *D*–*G* and *SI Appendix*, Fig. S3 *G*–*M*). In assessing the function of REV-ERBα in promoting NE-like cellular features, we observed that REV-ERBα OE significantly increased the number of cells with neurites-like protrusion. Its OE also significantly increased the nuclear expression of LP driver BRN2 and expression of NE marker SYP (Fig. 3 *H* and *I* and *SI Appendix*, Fig. S3 *N* and *O*). Together, these results demonstrated that elevated REV-ERBα promotes ARSIs resistance and aggressive cellular features that are associated with tumor LP.

### ARSI Reprograms REV-ERBα to Activate the LP Driver Network and LP Programs.

**Fig. 4.**
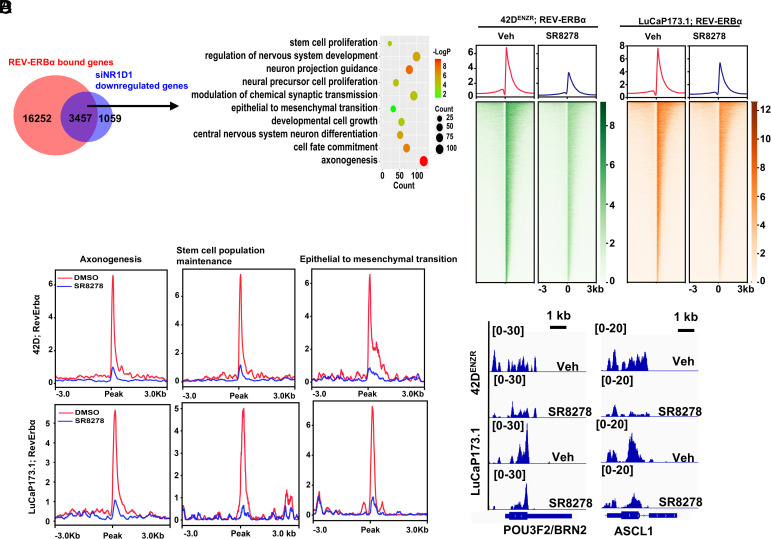
ARSI reprograms REV-ERBα to activate LP drivers and gene programs. (*A*) *Left*, venn diagram of the number of genes with REV-ERBα ChIP-seq peaks and expression significantly downregulated (>1.5-fold) by NR1D1 gene knockdown in 42D^ENZR^ cells. *Right*, GO analysis of the 3,457 genes was performed. 10 representative programs are shown in the bubble plot. (*B* and *C*) Heatmaps (*B*) and signal profiles (*C*) of REV-ERBα ChIP-seq signal intensity within ±3 kb around the peak center at genome wide or indicated LP programs in 42D^ENZR^ cells and LuCaP173.1 tumor cells treated with SR8278. (*D*) IGV snapshots of chromatin occupancies of REV-ERBα at chromatin regions of POU3F2 and ASCL1 in 42D^ENZR^ cells and LuCaP173.1 tumors treated as in (*B*).

Our findings that REV-ERBα plays a pivotal role in stimulating the LP programs promoted us to examine its functional mechanism. We thus performed REV-ERBα ChIP-seq with ARSI-resistant 42D^ENZR^ cells and found that a major proportion of REV-ERBα binding sites are located at promoter regions (*SI Appendix*, Fig. S4*A*). Integration of REV-ERBα bound genes with genes downregulated by REV-ERBα KD revealed that genes directly activated by REV-ERBα are significantly enriched in programs of axonogenesis (121 out of 460 genes in the program), stem cell proliferation (22 out of 64 genes), EMT (32 out of 153 genes), and developmental cell growth (56 out of 222 genes) (Fig. 4*A* and Dataset S2). They include all the major LP drivers as described in Fig. 2 *C*–*E*. Our additional ChIP-seq analysis with a de novo NEPC PDX tumor (LuCaP173.1) demonstrated that REV-ERBα ChIP-seq peaks in the PDX tumor are largely overlapping with the peaks in 42D^ENZR^ cells (*SI Appendix*, Fig. S4*B* and Dataset S2). The overlapped peaks-linked genes are significantly enriched in essentially the same LP programs as in 42D^ENZR^ cells (*SI Appendix*, Fig. S4*C* and Dataset S2). To examine the effects of REV-ERBα inhibition on its chromatin occupancy, we treated 42D^ENZR^ cells or the PDX tumors with its antagonist SR8278. The treatments strongly reduced genome-wide binding by REV-ERBα (Fig. 4*B*). Interestingly, the inhibition by SR8278 was much more pronounced at LP gene programs than genome-wide (Fig. 4 *C* and *D*; compare Fig. 4*B* with Fig. 4*C*).

As described above, REV-ERBα function is reprogrammed in t-NEPC cells. ARSI withdrawal can reverse ARSI-resistant cells to an AR-driven adenocarcinoma state and induce transcriptional reprogramming, due to restored AR canonical function (13, 43–45). Indeed, ENZ withdrawal abolished the expression of LP drivers BRN2 and ASCL1 and LP marker SYP but showed a modest effect on the expression of REV-ERBα, suggesting a lack of function by REV-ERBα in induction of the LP programs in the absence of ARSI (*SI Appendix*, Fig. S4*E*). To examine how REV-ERBα is reprogrammed, we cultured 42D^ENZR^ cells in the absence of ENZ for 7 d and then performed REV-ERBα ChIP-seq. We found that the ENZ withdrawal dramatically diminished REV-ERBα binding at LP gene programs. Instead, strong REV-ERBα occupancies were observed at genes primarily in programs of kinase signaling and lipid metabolism (*SI Appendix*, Fig. S4 *F*–*H* and Dataset S2). Together with the above ChIP-seq and RNA-seq data, these results suggest that ARSI reprograms REV-ERBα chromatin occupancy to directly activate the LP driver network and LP programs in NEPC.

### REV-ERBα Recruits BRD4 and p300 to Remodel Local Chromatin in Activation of the LP Genes.

**Fig. 5.**
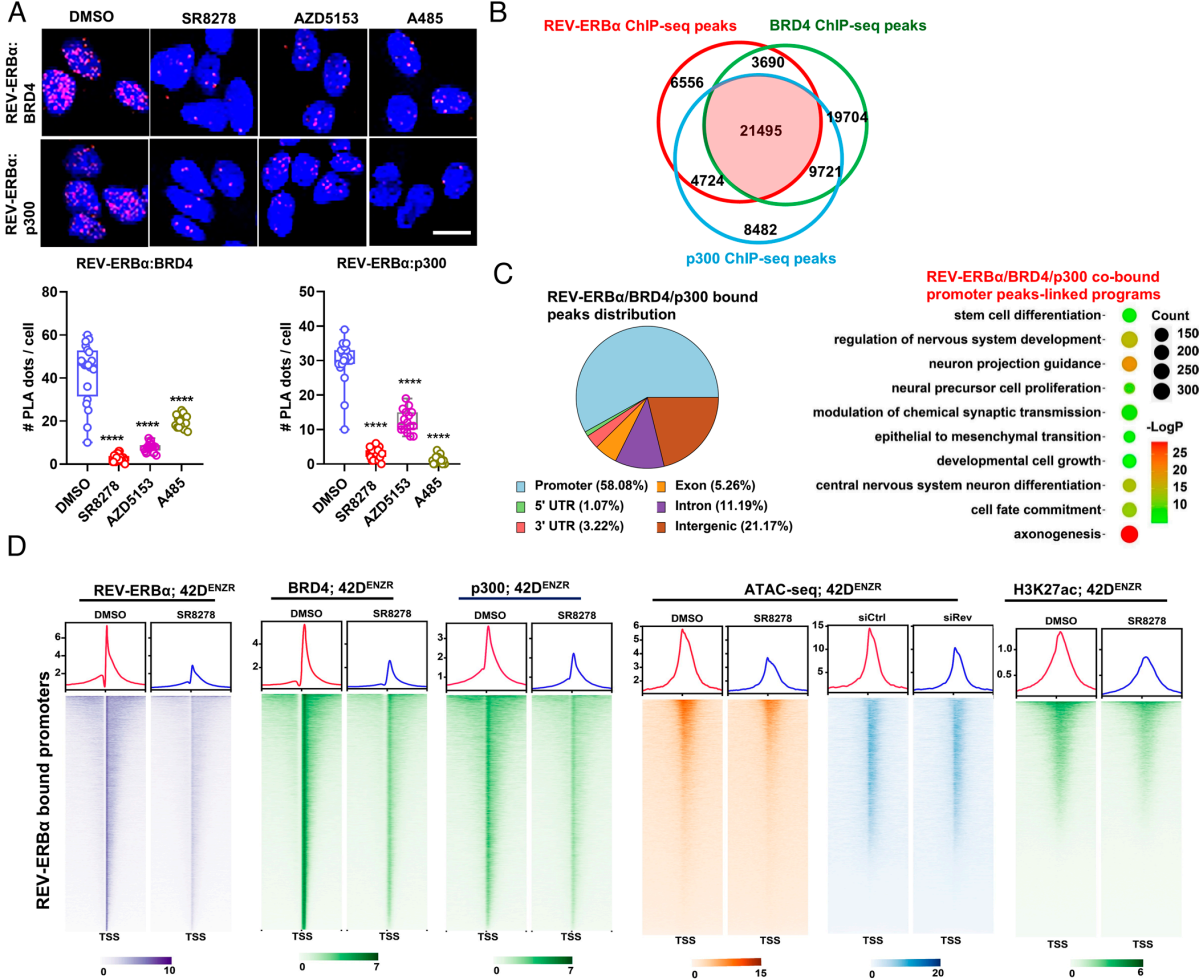
REV-ERBα recruits BRD4 and p300 to remodel local chromatin accessibility and to activate the LP genes. (*A*) PLA for protein interactions between REV-ERBα and coactivators BRD4 and p300 in 42D^ENZR^ cells. The cells were treated with 7.5 μM SR8278, 0.5 μM AZD5153, and 5 μM A485 for 24 h, and then Duolink assays were performed. (Scale bar, 50 μm.) Student’s *t* test. **P < 0.01, ***P < 0.001, ****P < 0.0001. (*B*) Venn diagram of the number of REV-ERBα ChIP-seq peaks, BRD4 ChIP-seq peaks, and p300 ChIP-seq peaks in 42D^ENZR^ cells. (*C*) *Left*, Peak distribution analysis of REV-ERBα, BRD4, and p300 cobound ChIP-seq peaks in *B*; *Right*, GO analysis of cobound peaks-linked gene programs in *B* (*Right*). (*D*) Heatmaps of REV-ERBα, BRD4, p300 binding peak intensity, chromatin accessibility, and H3K27ac signal within ±3 kb around REV-ERBα bound promoters in 42D^ENZR^ cells treated with 7.5 μM SR8278 or REV-ERBα KD for 24 h.

We previously found that in ARSI-sensitive cells and tumors, REV-ERBα switches its function from a repressor to an activator by association with epigenetic regulators BRD4 (Bromodomain-Containing Protein 4) and p300 (39). Indeed, in ARSI-resistant cells, REV-ERBα association with BRD4 and p300 was readily detected by PLA, and the association was strongly disrupted by REV-ERBα antagonist SR8278, BRD4 inhibitor AZD5153, and p300 acetylase inhibitor A485 (Fig. 5*A*). To examine whether BRD4 and p300 play a role in mediating the function of REV-ERBα, we performed ChIP-seq analysis of BRD4 and p300 in the ARSI-resistant cells. Remarkably, the majority of the three protein ChIP-seq peaks were overlapping (Fig. 5*B* and Dataset S2). The overlapped peaks are located primarily at gene promoter regions. Genes cobound by the three proteins are significantly enriched in the LP programs that are directly activated by REV-ERBα (Fig. 5*C* and Dataset S2), suggesting that BRD4 and p300 are involved in the activation function of REV-ERBα.

To further study the functional involvement of BRD4 and p300, we first examined the effects of REV-ERBα inhibition on the chromatin occupancies of BRD4 and p300. We found that REV-ERBα antagonist strongly diminished the bindings of BRD4 and p300 at LP gene promoters (Fig. 5*D* and *SI Appendix*, Fig. S5 *A* and *B*), indicating that BRD4 and p300 are recruited by REV-ERBα to the LP targets. We then examined the effects on local chromatin accessibility and found that REV-ERBα inhibition by either its antagonist or KD strongly reduced the local chromatin accessibility at the promoters of LP genes. Consistent with the functional involvement of p300, the inhibition also diminished the H3K27ac mark at the promoters (Fig. 5*D* and *SI Appendix*, Fig. S5 *A* and *B*). As shown in Fig. 2*G*, REV-ERBα OE alone strongly stimulated the expression of LP drivers. Consistently, we found that REV-ERBα OE strongly promotes BRD4 occupancy and local chromatin accessibility at promoters of LP driver ONECUT2 and ASCL1 (*SI Appendix*, Fig. S5*C*).

To study how BRD4 and p300 mediate the REV-ERBα function, we treated the cells with BRD4 inhibitor AZD5153, which blocks bromodomain association with acetylated histone, and the p300 acetylase inhibitor. We found that both inhibitors diminished the binding of REV-ERBα at promoters of LP genes (*SI Appendix*, Fig. S5 *D*–*F*). However, the binding inhibition was less potent than that of REV-ERBα antagonist as shown in Fig. 5*A*, suggesting that REV-ERBα binding to its target is partially facilitated by the functions of BRD4 and p300. Nevertheless, the BRD4 and p300 inhibitors strongly suppressed the mRNA and protein expression of major LP drivers and markers (*SI Appendix*, Fig. S5 *G* and *H*). Taken together, these results demonstrated that in ARSI-resistant cells, REV-ERBα recruits BRD4 and p300 to remodel local chromatin accessibility in activating the LP genes.

### REV-ERBα Facilitates Chromatin Occupancy of Known LP Drivers BRN2, ASCL1, and FOXA1.

**Fig. 6.**
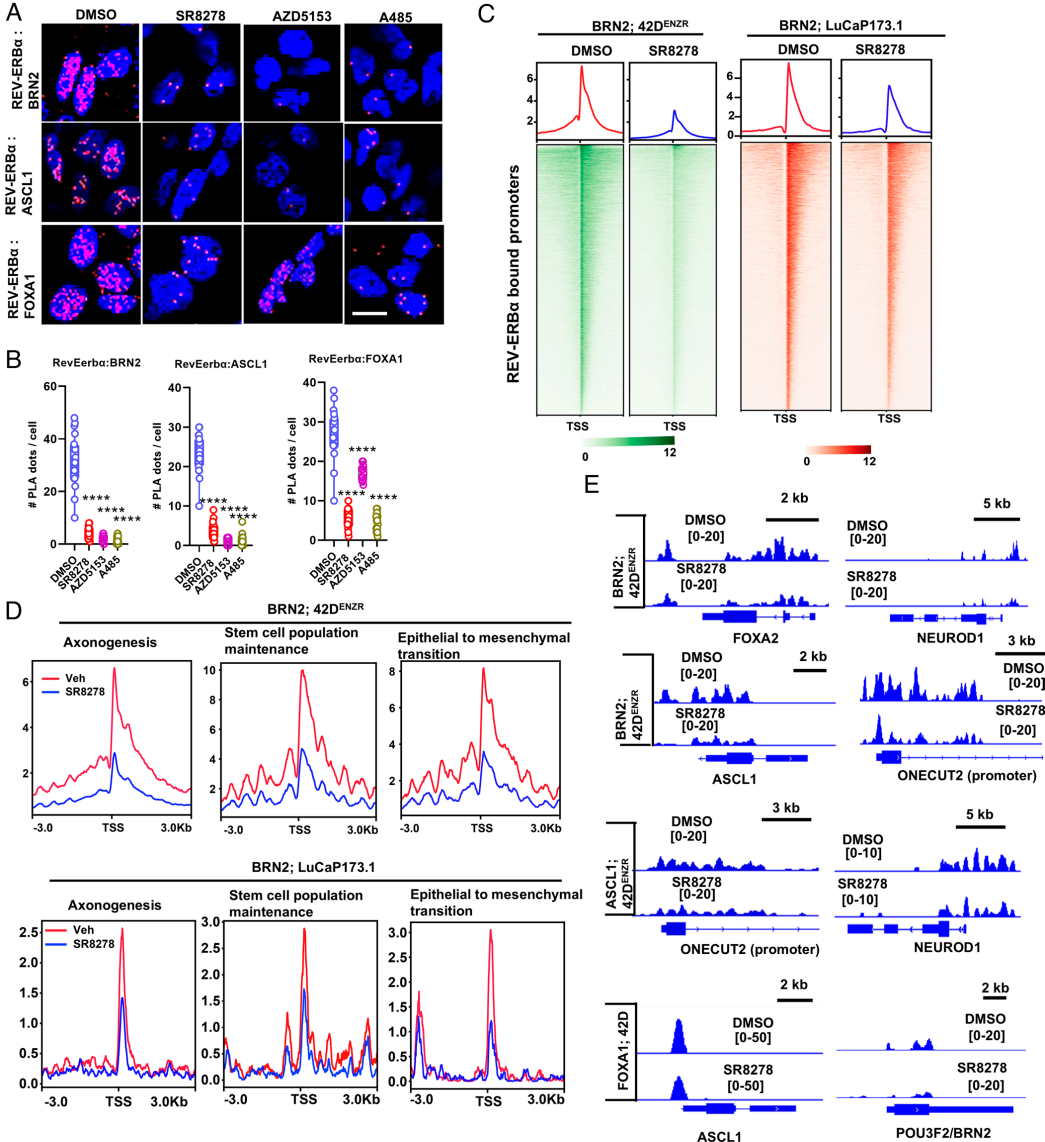
REV-ERBα facilitates chromatin occupancy of known LP drivers BRN2, ASCL1, and FOXA1. (*A* and *B*) PLA for protein interactions between REV-ERBα and LP drivers BRN2, ASCL1, and FOXA1 in 42D^ENZR^ cells. The cells were treated with 7.5 μM SR8278, 0.5 μM AZD5153, and 5 μM A485 for 24 h. (Scale bar, 50 μm.) Student’s *t* test. **P < 0.01, ***P < 0.001, ****P < 0.0001. (*C* and *D*) Heatmaps (*C*) and signal profiles (*D*) of BRN2 binding peak intensity within ±3 kb around REV-ERBα-bound promoters and LP programs in 42D^ENZR^ cells and LuCaP173.1 tumors treated with SR8278. (*E*) IGV snapshots of the ChIP-seq peaks at chromatin region of LP drivers in 42D^ENZR^ cells treated as in (*C*).

To further understand the functional mechanism of REV-ERBα, we assessed the potential involvement of other known LP drivers such as ASCL1, BRN2, and FOXA1. Our motif analysis of REV-ERBα binding sites over 200 bp regions revealed that in addition to REV-ER-like, half site (28.5% of target) and DR2 (2.1% of targets) motifs of ASCL1, FOXA1, and BRN2 are significantly enriched (*SI Appendix*, Fig. S6*A* and Dataset S2). Indeed, our PLA and coimmunoprecipitation (co-IP) analysis demonstrated strong associations between REV-ERBα and the other LP driver BRN2, ASCL1, and FOXA1 in ARSI-resistant cells. The associations were markedly disrupted by the REV-ERBα antagonist (Fig. 6 *A* and *B* and *SI Appendix*, Fig. S6*B*). Remarkably, the associations were also strongly disrupted by the inhibitors of BRD4 and p300, suggesting that REV-ERBα associations with the other LP drivers depend on the function of BRD4 and p300.

To examine whether REV-ERBα acts together with the known LP drivers, we performed BRN2, ASCL1, and FOXA1 cistrome analysis with cells treated by the REV-ERBα antagonist. As shown in *SI Appendix*, Fig. S6 *C* and *D*, over 75% of BRN2 peaks and over 50% of ASCL1 peaks are overlapped with REV-ERBα peaks at the promoter regions. Over 15% of FOXA1 peaks at genome-wide are overlapped with REV-ERBα peaks (*SI Appendix*, Fig. S6*C* and Dataset S2). As expected, genes linked to the overlapped peaks are significantly enriched in the LP programs (*SI Appendix*, Fig. S6*E* and Dataset S2). Treatment of cells with the antagonist for 24 h did not significantly alter the protein expression of the other LP drivers (*SI Appendix*, Fig. S6*B*). Remarkably, the antagonist treatment potently diminished the genome-wide binding of BRN2 in the treated cells (Fig. 6*C*). The marked inhibition can also be observed at the promoters of specific LP programs and drivers (Fig. 6 *D* and *E*). Similar inhibitory effects on BRN2 binding were also observed in the NEPC PDX tumors (Fig. 6 *C*–*E*). Although the antagonist effects on bindings of ASCL1 and FOXA1 were not as potent as for BRN2, apparent inhibition of ASCL1 occupancy at promoters and genome-wide binding by FOXA1 was observed in cells treated by the antagonist (*SI Appendix*, Fig. S6 *F* and *G* and Fig. 6*E*). Together, these results suggest that REV-ERBα facilitates chromatin occupancy of known LP drivers BRN2, ASCL1, and FOXA1, in addition to control of their expression.

### Pharmacological Inhibition of REV-ERBα Potently Suppresses NEPC Tumor Growth and LP Gene Programs In Vivo.

**Fig. 7.**
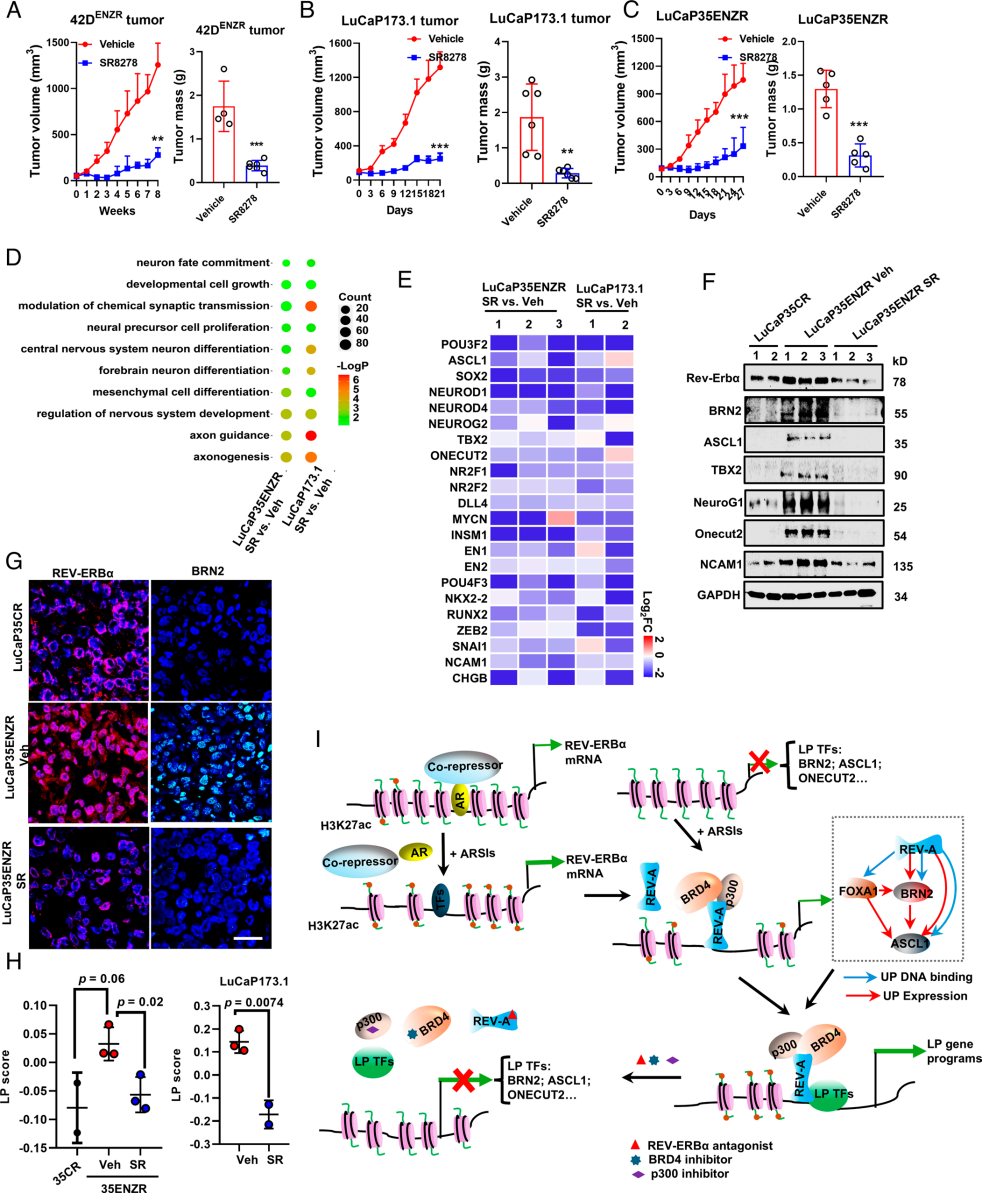
Pharmacological inhibition of REV-ERBα potently suppresses tumor growth and LP gene programs in vivo. (*A*–*C*) Mice bearing 42D^ENZR^ (*A*), LuCaP173.1 (*B*), and LuCaP35ENZR tumors (*C*) were treated, i.p., 5 times per week, with vehicle or 20 mg/kg SR8278 for indicated days. Tumor growth curves were plotted and tumor mass was measured at endpoint. Student’s *t* test. **P < 0.01, ***P < 0.001, ****P < 0.0001. (*D*) Bubble plots show the representative programs significantly enriched by GO analysis of genes down-regulated (>1.5-fold) in LuCaP35ENZR and LuCaP173.1 tumors treated with 20 mg/kg SR8278 (i.p.) for 10 consecutive days. (*E*) Heatmap of mRNA expression down-regulated in LuCaP35ENZR and LuCaP173.1 tumors treated with 20 mg/kg SR8278 detected by RNA-seq. (*F*) Intratumoral levels of proteins were detected by immunoblotting. (*G*) Representative images of IF staining of Rev-Erbα and BRN2 in LuCaP35CR and ENZR tumors treated with vehicle or SR8278. (Scale bar, 100 μm.) (*H*) Dot plot showing changes of LP signature scores for LuCaP35, LuCaP35ENZR, and LuCaP173.1 tumors before and after treated with ENZ, SR8278, or combination. (*I*) A graphic summary of a working model. See *Discussion* for details.

Given our results suggesting that REV-ERBα is a master regulator in ENZR cells, we next evaluated the therapeutic value of REV-ERBα inhibition using several ENZ-resistant NEPC models. First, in a t-NEPC model (42D^ENZR^ xenografts) and a de novo NEPC model (LuCaP173.1 PDX), intraperitoneal administration of SR8278 at 20 mg/kg potently blocked tumor growth, without discernable effects on the animal body weight (Fig. 7 *A* and *B* and *SI Appendix*, Fig. S7 *A*, *B*, and *D*). We then developed an AR-positive, ENZ-resistant PDX subline from LuCaP35 CRPC PDX model through consecutive treatments of the tumor-carrying mice with 20 mg/kg ENZ (*p.o.*) (see *SI Appendix*, *SI Materials and Methods* for details). To determine that LP programs were induced in the ENZR tumors, we first performed RNA-seq analysis. Consistent with the effects observed in 42D^ENZR^ cells, the same LP programs that are activated by REV-ERBα were significantly enriched in the ENZR tumors (*SI Appendix*, Fig. S7*E* and Dataset S3). The expression of REV-ERBα and the other LP drivers BRN2, ASCL1, NEUROG1, and ONECUT2 and NE markers CHGA and CHGB was markedly induced in ENZR tumors (*SI Appendix*, Fig. S7*F*). Significantly, treatment of mice carrying the LuCaP35ENZR tumors with SR8278 elicited a potent inhibitory effect with the tumor growth being largely blocked over the course of treatment (Fig. 7*C*). Moreover, over 50% of genes that were stimulated by ENZ were down-regulated by SR8278 in the ENZR tumors, indicating that a primary mechanism of action of REV-ERBα antagonist is suppressing ENZ-induced gene programs in vivo (*SI Appendix*, Fig. S7*G* and Dataset S3). GO analysis of tumor transcriptome revealed that SR8278 treatment significantly decreased expression of genes that are highly enriched in axonogenesis, developmental cell growth, neural precursor cell proliferation, and EMT in both treatment-induced ENZR tumors and de novo NEPC tumors (Fig. 7*D* and Dataset S3). Indeed, the expression of LP drivers BRN2, ASCL1, SOX2, NEUROD1/4, COUP-TF1/2, ONECUT2, MYCN, ZEB2, SNAI1, RUNX2, NKX2-2, and POU4F3 and NE markers (i.e., CHGA, NCAM1) was strongly inhibited by SR8278 treatment in almost all the tumors examined (Fig. 7*E* and Dataset S3). Consistent with RNA-seq data, the protein expression of REV-ERBα, the other LP drivers BRN2, ASCL1, ONECUT2, and NE marker NCAM1 was dramatically decreased by SR8278 treatment in the tumors as demonstrated by immunoblotting and immunofluorescence (IF) (Fig. 7*F* and *SI Appendix*, Fig. S7 *H*–*L*). Importantly, pharmacologic inhibition of REV-ERBα with SR8278 significantly reduced LP signature scores not only in LuCaP35ENZR but also in the de novo NEPC LuCaP173.1 tumors (Fig. 7*H*). These findings demonstrate that REV-ERBα activity is required for maintaining ARSI-induced LP in PDX models. Together, our findings from these animal experiments strongly suggest that pharmacological inhibition of REV-ERBα is effective in suppressing tumor LP programs and that REV-ERBα is an attractive therapeutic target for ARSI-resistant tumors.

## Discussion

Despite the recent progress in identification of major LP drivers including BRN2, ASCL1, and FOXA2 (25, 27, 46), relatively little is known about the mechanisms underlying the initial events such as ARSI induction of the drivers and their functional coordination in subsequent activation of the LP programs. In this study, we demonstrate that REV-ERBα, a TF of nuclear receptor/circadian regulator family, plays a crucial role in ARSI induction of a large number (more than 15) of established and putative LP driver TFs by directly activating their expression. Remarkably, REV-ERBα also facilitates the function of LP drivers such as ASCL1, BRN2, and FOXA1 through promoting their recruitment to LP genes. Thus, our findings strongly suggest that development of LP and ARSI resistance entails a coordinated induction of a network of LP drivers and functional cooperation of induced TFs and that REV-ERBα is an unexpected master regulator of the LP driver network.

Our findings highlight a critical role of REV-ERBα in the initiation of tumor LP. We observed that ARSI treatment rapidly induced REV-ERBα prior to the activation of canonical LP drivers, including BRN2, SOX2, and ASCL1, suggesting that REV-ERBα functions upstream in this regulatory cascade. Genetic ablation of REV-ERBα abolished ARSI-induced expression of these LP drivers and their associated transcriptional programs, while REV-ERBα OE alone was sufficient to prime adenocarcinoma cells toward a plastic state, even in the absence of ARSI. These observations indicate that REV-ERBα is not simply a downstream effector but acts as an initiating factor that establishes a permissive chromatin and transcriptional environment for LP induction. We propose a model in which REV-ERBα functions as an early-acting transcriptional switch, coordinating the initial induction of LP-driving networks that later consolidate lineage reprogramming. This mechanism provides insight into how prostate tumors acquire plasticity under ARSI pressure and positions REV-ERBα as a key molecular node in the earliest stages of LP initiation.

REV-ERBα appears to possess several unique attributes in acting as a master regulator of tumor LP. First, different from the other LP drivers such as ASCL1 and BRN2 which are significantly induced only in ARSI-treated tumor cells and thus function only in the resistant tumors, REV-ERBα expresses and plays very important roles in ARSI-sensitive adenocarcinomas by activating key tumorigenic programs including MAPK and PI3K-Akt signaling, cell cycle, and lipid metabolism (39). Remarkably, REV-ERBα expression is further increased by ARSI and plays a distinct role in ARSI-resistant tumor cells by activating the LP drivers and LP programs. Second, the function of REV-ERBα in facilitating chromatin occupancy at LP targets by the other LP driver TFs (e.g., ASCL1, BRN2, and FOXA1) is remarkable. Few TFs possess such activity (47). We recently found that REV-ERBα facilitates FOXA1 chromatin occupancy at non-AR target genes in ARSI-sensitive adenocarcinomas. Therefore, it is possible that such pioneer-like activity of REV-ERBα in ARSI-resistant tumors is “inherited” from the ARSI-sensitive tumor cells. Indeed, like what occurs in the sensitive cells, REV-ERBα recruits BRD4 and acetylase p300 to remodel local chromatin through increasing accessibility and H3K27ac mark in facilitating the chromatin occupancy by the LP drivers. Thus, ARSI triggers at least two major events in the initiation of LP process and ARSI resistance. One is the induction of master LP drivers such as REV-ERBα and the second is a genome-wide chromatin landscape reconfiguration which allows elevated REV–ERBα–BRD4–p300 complex to occupy newly accessible sites and induce the expression of a network of LP driver TFs (Fig. 7*I*). The network of TFs in turn stimulates the expression of other LP genes including their own. It is intriguing that the ARSI-exposed genomic regions contain noncanonical sequences for REV-ERBα such as the REV-RE half sites that we observed at the LP driver genes. It is tempting to speculate that REV-ERBα OE, as we observed in ARSI-mediated induction or our forced, ectopic expression, promotes its “sampling” of the new sites as observed in other pioneer factors (48–53) and/or stabilizes its occupancy at the targets for productive gene activation.

The third attribute of REV-ERBα is that it is a highly attractive therapeutic target for ARSI-resistant tumors such as NEPC. As shown in this study, its antagonist SR8278 is highly effective in blocking tumor growth of both t-NEPC and de novo NEPC. As expected, the expression of LP drivers and other LP program genes in the tumors is effectively suppressed. Importantly, the antagonist not only disrupts the association between REV-ERBα and its coactivators but also destabilizes REV-ERBα protein in the ARSI-resistant tumors. Future studies are warranted to further understand the initial events of LP development and to further develop inhibitors such as SR8278 for more effective treatment of ARSI-resistant tumors through blocking LP. Many studies including our study here demonstrate that anti-AR therapies, including Enzalutamide, Apalutamide, Bicalutamide, and Darolutamide, can induce LP in prostate adenocarcinoma, highlighting a potential adaptive response that may contribute to therapy resistance, suggesting that combining ARSIs with targeted interventions that block LP, such as inhibition of key regulators like REV-ERBα, may improve therapeutic outcomes and prevent ARSI resistance.

## Materials and Methods

Cell culture and siRNA transfection. All cells were grown in a humidified incubator with 5% CO2 at 37 °C. C4-2B cell line (54) was cultured in RPMI 1640 supplemented with 5% FBS (Gibico). CRPC 16D and t-NEPC 42D^ENZR^ cells were obtained from Amina Zoubeidi (University of British Columbia, Canada). These two cells were cultured in RPMI-1640 containing 10% FBS (Gibco), with the ENZ-resistant 42D^ENZR^ cell line supplemented with 10 μM ENZ (MCE, cat. #HY-70002) for all experiments unless otherwise stated. LAPC4 cells were kindly provided by Charles Sawyers (MSKCC, New York). All the cell lines used in this research were authenticated by STR profiling and regularly tested to be negative for *Mycoplasma*. For developing C4-2BEZNR and LAPC4ENZR cells, C4-2B and LAPC4 parental cells were cultured in the medium supplemented with 20 μM ENZ for 3 mo and the resistance of these cells to ENZ was validated by cell viability assay.

Information of the compounds, Methods of siRNA transfection, Clinical tumor gene expression analysis, Plasmid constructs and generation of stable transfectants, Cell viability and cell growth assays, qRT-PCR and Western blotting analysis, Migration and invasion assays, Spheroid formation assay, Flow cytometry, IF, ChIP-seq, RNA-seq, and ATAC-seq and their data analyses, co-IP, proximity ligation assay (PLA), Establishment of ENZ-resistant PDX tumor models and animal experiments, Cryosectioning and IF are also provided in *SI Appendix*, *SI Materials and Methods*.

## Supplementary Material

Appendix 01 (PDF)

Dataset S01 (XLSX)

Dataset S02 (XLSX)

Dataset S03 (XLSX)

## Data Availability

All raw data and processed information for RNA-seq (GSE295834) (55), ChIP-seq (GSE295844) (56), and ATAC-seq (GSE295843) (57) in this study have been deposited in the Gene Expression Omnibus (GEO). All other data are included in the manuscript and/or supporting information.
