# Supplementary material for: Circadian regulator REV-ERBα is a master regulator of tumor lineage plasticity and an effective therapeutic target
Source: Proc Natl Acad Sci U S A. 2025 Nov 13;122(46):e2513468122. doi: 10.1073/pnas.2513468122 (PMC12646269; doi:10.1073/pnas.2513468122)
Supplement: Supplementary file 1 — Appendix 01 (PDF) [file pnas.2513468122.sapp.pdf]

Supporting Information for

Circadian regulator REV-ERBα is a master regulator of tumor lineage plasticity and an effective therapeutic target

Xiong Zhang, Yatian Yang, Hongye Zou, Demin Cai, Eva Corey, Amina Zoubeidi, Su Hao Lo, Ai-Ming Yu, Ronald M Evans and Hong-Wu Chen

This PDF file includes:

Supporting text

Figures S1 to S7

Figure legends S1 to S7

Tables S1 to S3

Legends for Dataset S1 to S3

SI References

Other supporting materials for this manuscript include the following:

Datasets S1 to S3

Supporting Information Text

SI materials and methods

Clinical tumor gene expression analysis

The Beltran datasets for 34 adenocarcinoma and 15 t-NEPC samples were downloaded from cBioPortal website (https://www.cbioportal.org/study/summary?id=nepc_wcm_2016). GSE21032, GSE48403, GSE197781 and GSE 181462 datasets were downloaded from the Gene Expression Omnibus (GEO) database. Based on the gene profile across the samples, the Pearson correlation metric was computed between each gene (i.e., NR1D1 and ASCL1) in R software and visualized using ggplot2 function.

Chemicals

SR8278 (purity > 98%) were synthesized by WuXi AppTec. Other chemicals (purity > 98%) are from MCE and Selleck unless indicated otherwise.

siRNA transfection

siRNAs were synthesized by Dharmacon (Cambridge, UK). The siRNA sequences against REV-ERBα gene NR1D1 were GCTGGCATGTCCTATGAACAT for siRev-1, and GCGCTTTGCTTCGTTGTTCAA for siRev-2. siRNA sequences against ASCL1 gene were GCGCGGCCAACAAGAAGAUGAGUAA for siASCL1-1, and UUACUCAUCUUCUUGUUGGCCGCGC for siASCL1-2. siRNA sequences against FOXA1 gene were GGACUUCAAGGCAUACGAA for siFOXA1-1, and GUGUAGACAUCCUCCGUAU for siFOXA1-2. siRNA sequences against BRN2 gene POU3F2 were CTGGACGGGCGTCTGCAC for siBRN2-1, and GACCCGCACTCGGACGAGGAC for siBRN2-2. siRNA sequence CAGTCGCGTTTGCGACTGG was used as non-targeting control. Transfection of siRNAs was performed with OptiMEM (Invitrogen, cat. #11058021) and Dharmafect1 (Dharmacon, cat. #T-2001-02), as described (1) following the manufacturer's instructions.

Plasmid constructs and generation of stable transfectants

For REV-ERBα/NR1D1 overexpression, human NR1D1 cDNA as mentioned (1) was sub-cloned into a modified pLX304 vector with a V5 tag at the receptor N terminus. Lentiviral particles were produced using 293T cells with co-transfection of the above lentivirus vectors, psPAX2 and pMD2.G in 10-cm dishes. To generate REV-ERBα/NR1D1 overexpression stable transfectants, cells were infected with virus-containing supernatants in the presence of 10 ng/mL polybrene (sigma, TR-1003-G) for 6 h and followed by the selection under 20 μg/mL blasticidin for a consecutive two weeks before cells were replated for individual colony of growth in the selection medium. Cells from different colonies were then isolated and expanded as stable clones.

sgRNAs targeting human NR1D1 gene were designed using CRISPOR design software (http://crispor.tefor.net/). Oligos corresponding to the sgRNAs were synthesized and cloned into lentiCRISPR v2 vectors following lentiCRISPRv2 and lentiGuide oligo-cloning protocol (Addgene, plasmid#52961). The sgRNA sequences are as follows: sgGFP, GGGCGAGGAGCTGTTCACCG; sgNR1D1, CAAGACCCGGCTCGCTCCTT. Lentiviral particles were produced in 293T cells as previously described (1). Prostate cancer cells were seeded into 6 well plates at a density of 2×10^5 cells per well. Twenty-four hours later, 1 ml of virus-containing supernatant mixed with 1 mL fresh medium containing 20 ng/mL of polybrene was added to the cells. After 6 h of incubation, the medium was changed to regular medium and maintained in the selection medium in the presence of 0.5 μg/mL Puromycin for one week before cells were replaced for individual colony of growth in the selection medium. Cells from different colonies were then isolated and expanded as stable NR1D1 KO clones.

Cell viability and cell growth assays

For cell viability assay, prostate cancer cells were seeded in 96-well plates at 2000 cells per well in a total volume of 100 μL media with 3 replicates. Then another 100 μL media with diluted compounds were added into each well next day. After 4 days of incubation, the medium was discarded and 50 μL Cell-Titer Glo reagents (Promega, cat. #G9243) were added and incubated for 5 min before luminescence was measured on a GLOMAX microplate luminometer (Promega), according to the manufacturer's instructions. For cell

growth assay, prostate cancer cells were seeded into 6 well plates at a density of 2×10^5 cells per well with triplicates. Twenty-four hours later, cells were transfected with siRNA or treated with compounds as indicated. Live cells were counted with a Cell Counter (Thermo Fisher Countess II) in the presence of trypan blue reagent. The above assays were performed in triplicates and entire experiments were repeated three times.

qRT-PCR and immunoblotting analysis

Total RNA was isolated from cells or xenograft tumors using TRIzol regent (Invitrogen, cat. 15596026). One µg of total RNA was reverse transcribed to cDNA using qScript cDNA SuperMix (Quantabio, cat. #95047-025). Then qRT-PCR was performed using SYBR Green master mix (Applied Biosystems, cat. A25742) and gene specific primers. The PCR was run on a CFX96 connect Real-Time PCR system (Biorad). GAPDH gene transcript was used for normalization. The 2-ΔΔCT method was used to obtain the relative quantifications. The experiments were performed at least three times with data presented as mean values ± SD. The primers are shown in SI Appendix, table S1. Cell or tissue lysates were analyzed by immunoblotting with antibodies specifically recognizing indicated proteins. The antibodies and dilution ratio used are shown in SI Appendix,Table 2.

Migration and invasion assays

Twenty four-well plates with 8 µm pore size inters were used (Corning, cat. #3422). For invasion assays, inserts were precoated with 100 µL diluted Matrigel (BD Biosciences, cat. # 354324) at 20% dilution. Prostate cancer cells were seeded into 6 well plates at a density of 2×10^5 cells per well with triplicates. Twenty-four hours later, cells were transfected with siRNA or treated with compounds as indicated for 24 h. Single cell suspensions were made using serum-free medium. 100 µL of cell suspensions containing 1 x 10^5 were plated onto each insert. 600 µL medium with 10% FBS was added in the lower chamber to induce cell motility. 24 h later for migration assays and 48 h later for invasion assays, the membrane was fixed in cold 70% ethanol and stained with 0.5% crystal violet in methanol. Cells and gel material on the upper chamber were removed. The migrated or invaded cells were imaged under a phase-contract microscope using the 10x magnification lens. Cells form randomly 5 chosen fields were counted and analyzed in Graphpad software.

Spheroid formation assay

Single cell suspensions of REV-ERBα OE stable cells (500 cells/mL) or 42D cells (2000 cells/mL) were plated on ultra-low attachment plates and cultured in serum-free Advanced DMEM/F-12 medium (Gibco, cat. #12634010), supplemented with 2% B27 (Thermo, cat. #A3582801), 20 ng/mL human epidermal growth factor (hEGF) (Sigma-Aldrich, cat. #E5036), 10 ng/mL human insulin (Invitrogen, cat. RP-10908) and 10 ng/mL basic fibroblast growth factor (PeproTech, cat. #100-18B) for 5 to 10 days. Tumor spheres were visualized and counted under a phase-contract microscope using the 10x magnification lens.

Flow cytometry

Treated cells were dissociated in dissociation buffer (3 m MEDTA in 1 x PBS) at 37 °C for 10 min. Cells then were harvested using cell scrapers and filtered through a 40-µm nylon cell strainer. Single cell suspensions were pelleted at 500 x g and re-suspended in flow cytometry buffer (3 mM EDTA, 0.5% BSA in 1 x PBS) with CD44, APC-conjugated (Biolegend, cat. #397506, dilution 1:20) and CD133, PE-conjugated (BD, cat. 566593, dilution 1:20) for 1 h at 4 °C. Isotype antibodies were used as negative control. Cells were washed 2 times with flow cytometry buffer and acquired on a FACS (BD Biosciences). Data was analyzed using FlowJo software (version 10.4.2).

Immunofluorescence (IF)

Forty-eight hours before staining, NR1D1 OE cells were seeded onto 8-well chamber slides (Lab-Tek II, cat. #154534) at a density of 5 x 10^4 / well. Cells were fixed in 4% paraformaldehyde (Thermo Fisher, cat. 30525-89-4) for 15 min and permeabilized in 0.2% Triton X-100 (Thermo Fisher, cat. A16016-AE) for 10 min at RT. Permeabilized cells were washed twice with PBST (PBS supplemented with 0.1% Tween-20) and blocked with blocking buffer (2% goat serum in 1 x PBS) for 1 h. Cells were incubated with primary antibodies REV-ERBα (cell signaling technology, cat. #13418, dilution 1:200), BRN2 (Santa Cruz, cat. Sc-

393334, dilution 1:200) and SYP (eBioscience, cat. #14-6525-82, dilution 1:200) at 4 °C overnight. Cells were then washed 3 times with PBST (0.1% Tween-20 in 1 x PBS) and incubated in the secondary antibody, goat anti-Rabbit IgG (H+L) Texas Red (Invitrogen, cat. #T-2767, dilution 1:200) and goat anti-Mouse IgG (H+L) Alexa Fluor 488 (Invitrogen, cat. #A-11017, dilution 1:200), for 1 h at RT. The nuclear were labelled by Hoechst33342 (ThermoFish, cat. #H3570). Cells were mounted on slides using anti-Fade Fluorescence Mounting Medium (Abcam, cat. #AB104135). Fluorescent images were taken under 20 x objective using Zeiss confocal microscope. The stain intensity form randomly chosen cells were counted and analyzed in Graphpad software.

Protein interaction analyses

For co-immunoprecipitation (co-IP) experiments, $42D^{ENZR}$ cells stably expressing NR1D1-V5 were growing in 10 cm dishes and incubated with BRD4 inhibitor AZD5153, p300 inhibitor A485 and different concentrations of inhibitor SR8278 for 24 h. Then cells were lysed with 400 µL lysis buffer (10 mM HEPES, pH 7.9, 10 mM KCl, 0.1 mM EDTA, 0.4% NP-40, and protease inhibitor cocktail) on ice for 15 min. Then nucleus was collected after centrifuging for 30 s at 3000 × g at 4 °C. Nuclear proteins were isolated by incubating the nucleus with 300 µL extraction buffer (20 mM HEPES, pH 7.9, 0.4 M NaCl, 1 mM EDTA, and protease inhibitor cocktail) for 30 min in a shaker at 1000 rpm at 4 °C. After diluted with dilution buffer (20 mM HEPES, pH 7.9, 1 mM MgCl2, 0.5% NP-40, 1 mM EDTA, and protease inhibitor cocktail) at a ratio of 1:2, nuclear proteins were incubated with either 50 µL Anti-V5-tag mAb-Magnetic Beads (MBL, M215-11) or 50 µL Mouse IgG1 (isotype control)-Magnetic Beads (MBL, M075-11) overnight at 4 °C. Immunoprecipitation beads were washed with wash buffer (50 mM Tris-HCl, pH7.5, 200 mM NaCl, 5 mM EDTA, and 1% Triton X-100) for 3 times, and immunoprecipitated proteins were eluted by 1× protein loading buffer. Then the eluted proteins were subjected to western blotting analysis.

For Proximity Ligation Assays, $42D^{ENZR}$ cells were seeded onto 8-well chamber slides (Lab-Tek II, cat. #154534) at a density of 5 x 10^4 / well and treated with 7.5 µM SR8278, 0.5 µM AZD5153, 5 µM A485 or vehicle for 24 h. The cells were then fixed with 4% paraformaldehyde (Thermo Fisher, cat. 30525-89-4) for 15 min and permeabilized in 0.2% Triton X-100 (Thermo Fisher, cat. A16016-AE) for 10 min at RT. Permeabilized cells were washed twice with PBST (PBS supplemented with 0.1% Tween-20) and blocked with blocking buffer for 1 h. Primary antibodies REV-ERBα (cell signaling technology, cat. #13418, dilution 1:500), BRD4 (cell signaling technology, cat. #63759, dilution 1:200), p300 (Active Motif, cat. 61401, dilution 1:200), BRN2 (Santa Cruz, cat. Sc-393334, dilution 1:200), ASCL1 (R&D systems, cat. AF2567, dilution 1:200) and FOXA1 (Active Motif, cat. #39837, dilution 1:200) were used to stain the cells overnight at 4 °C before cells were incubated with secondary antibodies for 1 h at 37 °C. Cells were then incubated with ligation buffer for 30 min and amplification buffer for another 100 min at 37 °C. Hoechst33342 (ThermoFish, cat. #H3570) were used to label nuclear. Imagine the PLA signals were done using a confocal microscope (Zeiss LSM780, USA) under 20 x magnification lens. The PLA dots in cells from randomly chosen fields were counted and analyzed in GraphPad software.

RNA-seq and data analysis

Total RNA was isolated from cultured cells or frozen tissues using TRIzol regent (Invitrogen, cat. #15596026). Library constructions were performed using the NEBnext Ultra ii Straned RNA Library Prep Kit, and sequencing was performed on an Illumina NextSeq 500 system (50 x 50 bp paired end reads). Data were trimmed using trim-glare and the resultant read sequences were aligned to the hg19 human reference genome using bowtie2 aligner. Aligned reads in Bam format were counted using HTSeq and transformed into FPKM in R software. Genes were ranked based on fold changes (FC) and genes with |FC| > 1.5 were subjected to Gene Ontology (GO) analysis in R using clusterProfiler package. Representative GO pathways were displayed using ggplot2 function. Signature scores of LP for each sample in Beltran prostate, 16D, $42D^{ENZR}$ cells, LuCaP35CR, LuCaP35ENZR and LuCaP173.1 tumors were generated with R “GSVA” package using “GOBP_NEURON_DIFFERENTIATION” gene set from GSEA database. Based on the signature scores and the gene profile across the samples, the Pearson correlation metric was computed between NR1D1 and each LP signature scores using the “cor” function in GraphPad software.

ChIP-seq and data analysis

REV-ERBα, H3K27ac, BRD4, p300, BRN2, ASCL1 and FOXA1 ChIP assays were performed with $42D^{ENZR}$ cells. 5 x 10^7 $42D^{ENZR}$ cells were treated with 7.5 μM SR8278, 0.5 μM AZD5153, 5 μM A485, vehicle or transfected with siRNAs targeting NR1D1/REV-ERBα or POU3F2/BRN2 for 24 h. Cells were fixed with 1% formaldehyde at room temperature for 8 min, and subsequently quenched with 0.125 mol/L glycine for another 8 min. Cells were washed with cold PBS and suspended with lysis buffer (50 mmol/L HEPES, pH 8.0, 140 mmol/L NaCl, 1 mmol/L EDTA, 10% glycerol, 0.5% NP-40, and 0.25% Triton X-100). Cell pellets were then resuspended in washing buffer (10 mmol/L Tris, pH 8.0, 1 mmol/L EDTA, 0.5 mmol/L EGTA, and 200 mmol/L NaCl), washed, and resuspended in shearing buffer (0.1% SDS, 1 mmol/L EDTA, pH 8, and10 mmol/L Tris-HCl, pH 8) before sonication. Sonication was performed using Covaris E220 following the manufacturer's instruction. Soluble fractions of sheared chromatin were immunoprecipitated by magnetic protein G beads coated with specific antibodies at 4 °C overnight. Chromatin-captured beads were washed five times with LiCl wash buffer (100 mmol/L Tris, pH 7.5, 500 mmol/L LiCl, 1% NP-40, and 1% sodium deoxycholate), and one time with TE buffer (10 mmol/L Tris, pH 7.5 and 0.1 mmol/L EDTA) before the chromatins were eluted by elution buffer (1% SDS and 100 mmol/L NaHCO3). The eluted DNA was incubated with RNase A for 30 min at 37 °C, followed by overnight incubation at 65 °C for reverse crosslinking. After proteinase K treatment, ChIP DNA was purified using PCR purification kit (Qiagen, #28104). Purified ChIP DNA was then used for ChIP-qPCR analysis and library generation. Libraries were prepared, analyzed with the Bioanalyzer 2100 (Agilent) and sequenced in either single-end (BGI, Hong Kong, China) or paired-end (Novogene, USA) 50-bp mode on Illumina Sequencers.

For REV-ERBα ChIP-seq and BRN2 ChIP-seq with LuCaP173.1 PDX tumor tissues, the tissues were dissected from mice that were treated with 20 mg/kg SR8278 (i.p.) for 10 days before harvested. The dissected tissues were first snap-frozen in liquid nitrogen and then thawed on wet ice before they were homogenized using a micro-tube homogenizer with disposable pellet pestles (Sigma, cat. #Z359955). Tissue homogenates were filtered through a 40-μm nylon cell strainer. Single cells were pelleted and resuspended in ice-cold PBS and subjected to ChIP-seq procedures as above.

ChIP-seq validated antibodies against REV-ERBα (Proteintech, cat. #14506-1-AP, 5 μg; Cell signaling technology, cat. #13418, 5 μg), BRN2 (Genetex, cat. #GTX114650, 5 μg), BRD4 (Abcam, cat. #ab272042, 5 μg), p300 (Abcam, cat. #ab14984, 5 μg), ASCL1 (Abcam, cat. #556604, 5 μg), FOXA1 (Abcam, cat. #ab23738, 5 μg) and H3K27ac (Diagenode, cat. #C15410196, 2 μg), were used as indicated in Table S1.

Single-end or paired-end ChIP-seq fastq files were trimmed using Trim Galore v0.6.10 (https://anaconda.org/bioconda/trim-galore) and reads were aligned to the hg19 human genome with Bowtie2 v 2.5.1 (https://anaconda.org/bioconda/bowtie2). PCR duplicates in BAM files were removed with samtools mardup function. Significant ChIP-seq peaks were called using MACS2 (v 2.2.7.1) using a ChIP input file as a control with P-value < 0.05 in narrow peak mode (for REV-ERBα, BRN2, ASCL1, p300 and FOXA1) or broad peak mode (for BRD4 and H3K27ac). The heatmaps and binding profiles of ChIP-seq data were generated by deeptools program using default parameters. The shared and unique peaks between various ChIPseq and ATACseq samples are generated using bedtools. The visualization of binding profiles at chromatin were displayed using IGV. For differential binding analysis, normalized binding intensities and log2FC for each peak were generated by Manorm algorism. Because multiple peaks may be annotated with the same gene, the peak with highest absolute value log2FC for each gene was kept for GSEA and GO analyses using the same parameters as RNA-seq analysis. Representative GO and GSEA pathways were displayed using ggplot2 function.

ATAC-seq and data analysis

ATAC-seq experiments were performed using $42D^{ENZR}$ cells. 50,000 variable cells were used for transposase-accessible chromatin experiments following the procedures as described (2). ATAC-seq data analysis was performed using nf-core ATAC-seq pipeline with default parameters. Deeptools, bedtools, IGV and Manorm were used for the downstream analysis as described in ChIP-seq analysis.

Establishment of ENZ-resistant PDX tumor models and animal experiments

The compounds enzalutamide and SR8278 were fully dissolved in a formulation of 15% Cremophor EL, 82.5% PBS, and 2.5% DMSO. Nude mice and SCID C.B-17 mice were purchased from Envigo. All mouse experiments were conducted under animal protocols approved by Institutional Animal Care and Use

Committee (IACUC) of University of California, Davis. LuCaP35ENZR tumors were derived from LuCaP35CR as previously reported (3). Castrated mice carrying LuCaP35CR tumors at approximately 100 mm^3 were treated with 20 mg/kg ENZ (p.o.) 5 times per week, for 45 days before the tumor grew to approximately 1000 mm^3. The tumor tissues were dissected and reimplanted to additional castrated mice and mice were treated as above for another 45 days before the re-grown tumors were harvested and reimplanted for the experiments.

To generate $42D^{ENZR}$ xenografts, mice were subcutaneously injected with 2 x 10^6 of $42D^{ENZR}$ cells per one site-right flank. When the tumors reached approximately 100 mm^3, five to six mice were randomized to treatment groups. To establish xenograft tumor models for the treatments, LuCaP173.1 and LuCaP35ENZR fresh tumors were isolated from mice and propagated by inserting ~2 mm3 into the right flank of each mouse. To achieve statistical significance, five to six mice were randomized to different groups when the tumors reached approximately 100 mm^3. Mice were then treated daily with SR8278 (20 mg/kg, i.p.) or vehicle for around 3 weeks depending on the model. For $42D^{ENZR}$ and LuCaP35ENZR tumors, all the mice were treated with 20 mg/kg ENZ (p.o.) to keep the resistant features. Tumor volumes were measured by using calipers with volume calculated using Eq. (1):

Tumor volumes = (Length × Width2)/2 (1).

Cryosectioning and Immunofluorescence (IF)

Fresh samples of prostate PDX tissues were cut into 0.5 cm in diameter and snap frozen using Tissue-OCT Compound (Sakura Finetek, MPSMK-981385). OCT-embedded tissues were sectioned into 5-15 μm thick pieces using a cryostat (Leica CM 1950, Netherlands) at −20 °C. The slides were fixed with 4% paraformaldehyde solution (ThermoFisher, J19943.K2), permeabilized with permeabilization buffer (0.2 % Triton™ X-100 in PBS) and blocked by blocking buffer (10 % FBS in PBS). Then blocked slides were incubated with primary antibodies diluted with blocking buffer overnight at 4 °C and fluorescently labeled secondary antibody dilutions at RT for another 1 h. Hoechst33342 (ThermoFish, H3570) were used to label the nuclear. Images were acquired using Zeiss LSM780 Confocal harboring 405-, 488-, 561-, and 633-nm lasers. Image analysis was done using ImageJ software. The antibodies and dilution ratio used are shown in SI Appendix,Table 2.

AR status

The AR status across our models were listed in SI Appendix,Table 3.

Statistical analysis

Cell culture experiments were conducted with triplicates. GraphPad Prism 7 was used for computing P values and statistical significance. Data were presented as mean ± standard deviation (mean ± SD). Differences between the treatments were made by Student's paired t test and $P < 0.05$ was considered statistically significant.

Data accessibility: All raw data and processed information for RNA-seq (GSE295834), ChIP-seq (GSE295844), and ATAC-seq (GSE295843) in this study have been deposited in the Gene Expression Omnibus (GEO).

Fig.S1

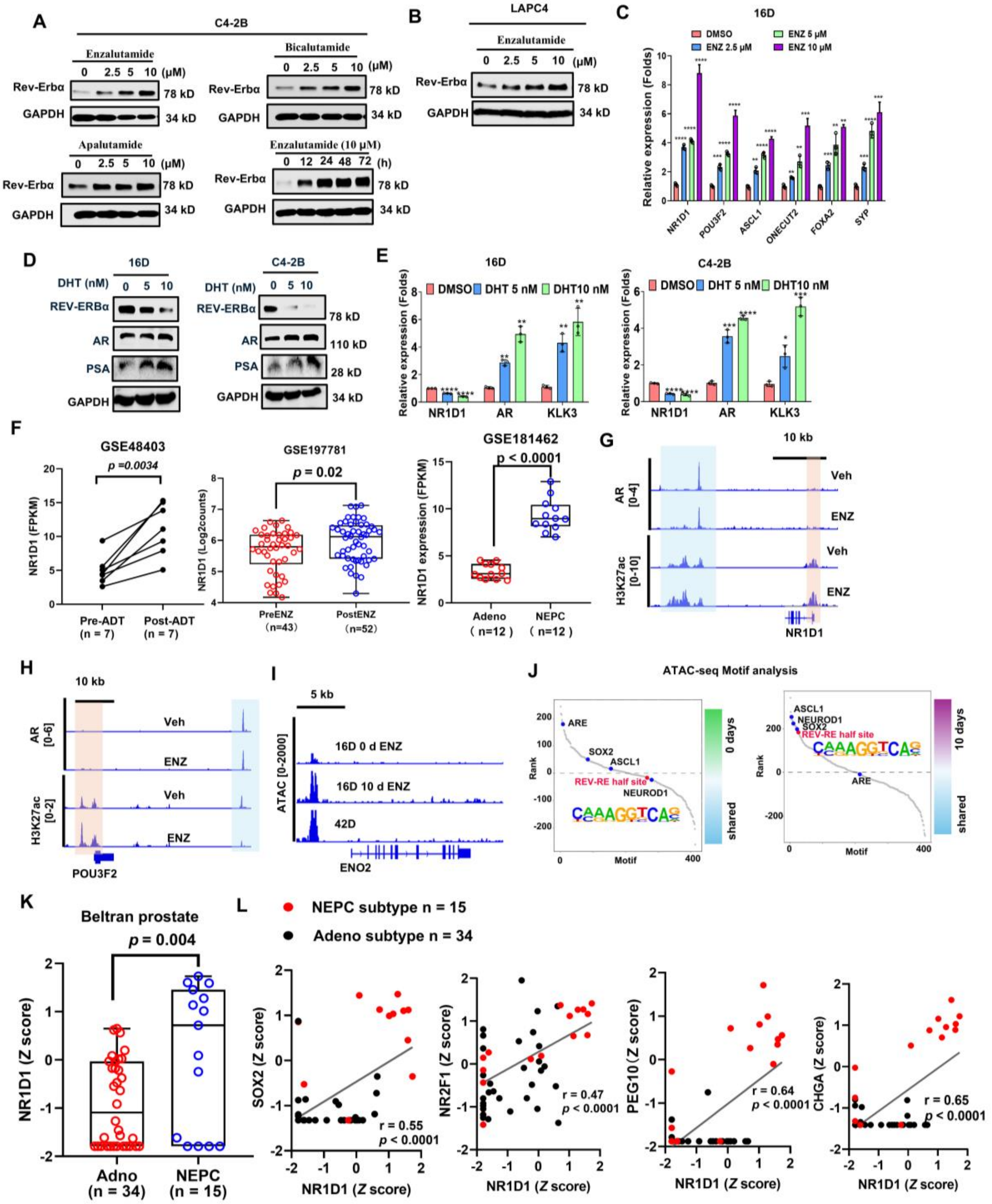
A
C4-2B
Enzalutamide
0 2.5 5 10 (μM)
Rev-Erbα
78 kD
GAPDH
34 kD
Bicalutamide
Apalutamide
Enzalutamide (10 μM)
0 12 24 48 72 (h)
B
LAPC4
C
16D
DMSO
ENZ 2.5 μM
ENZ 5 μM
ENZ 10 μM
Relative expression (Folds)
NR1D1
POU3F2
ASCL1
ONECUT2
FOXA2
SYP
D
16D
C4-2B
DHT (nM)
0 5 10
REV-ERBα
AR
PSA
110 kD
28 kD
E
DHT 5 nM
DHT10 nM
KLK3
F
GSE48403
p =0.0034
NR1D1 (FPKM)
Pre-ADT
(n = 7)
Post-ADT
(n = 7)
GSE197781
p = 0.02
NR1D1 (Log2counts)
PreENZ
(n=43)
PostENZ
(n=52)
GSE181462
p < 0.0001
NR1D1 expression (FPKM)
Adeno
(n=12)
NEPC
(n=12)
G
10 kb
AR [0-4]
H3K27ac [0-10]
Veh
ENZ
H
AR [0-6]
H3K27ac [0-2]
POU3F2
I
5 kb
ATAC [0-2000]
16D 0 d ENZ
16D 10 d ENZ
42D
ENO2
J
ATAC-seq Motif analysis
Rank
Motif
ARE
SOX2
ASCL1
REV-RE half site
NEUROD1
CAAAGGTCA
0 days
10 days
shared
K
Beltran prostate
p = 0.004
NR1D1 (Z score)
Adno
(n = 34)
NEPC
(n = 15)
L
NEPC subtype n = 15
Adeno subtype n = 34
SOX2 (Z score)
r = 0.55
p < 0.0001
NR2F1 (Z score)
r = 0.47
p < 0.0001
PEG10 (Z score)
r = 0.64
p < 0.0001
CHGA (Z score)
r = 0.65
p < 0.0001
NR1D1 (Z score)

Fig.S1 Elevated expression of AR-suppressed NR1D1/ REV-ERBα is associated with tumor LP. A Immunoblotting analysis of the expression of REV-ERBα in C4-2B cells (**A**) and LAPC4 cells (**B**) treated with indicated doses and times of ARSIs. **C** q-RT PCR analysis of expression of NR1D1 mRNA and lineage plasticity (LP) drivers POU3F2/BRN2, ASCL1, ONECUT2, FOXA2 and neuroendocrine marker SYP in CRPC 16D cells treated with indicated concentrations of ENZ for 96 h. **D-E** Immunoblotting (**D**) and q-RT PCR (**E**) analysis of expression of NR1D1 mRNA in CRPC 16D and C4-2B cells treated with indicated concentrations of Dihydrotestosterone (DHT) for 24 h. AR and its target gene KLK3 and its encoded protein PSA is used as an indicator of DHT activity. **F** The expression of NR1D1 in patients before and after ARSI abiraterone (GSE48403), ARSI ENZ (GSE197781, GSE181462). **G-H** IGV snapshots of chromatin occupancies of AR and H3K27ac signal at chromatin regions of NR1D1 (**G**) and LP driver POU3F2/BRN2 (**H**) in C4-2 cells treated with ENZ from GSE136128. **I** IGV snapshot displays changes of chromatin accessibility at neuroendocrine marker ENO2 chromatin regions in 16D cells treated with 10 μM ENZ for 10 days and its ENZR subline $42D^{ENZR}$ cells. **J** Transcription factor (TF) binding motifs surrounding accessible chromatin in unique vs. shared regions, ranked based on differential *p*-value. Data are from GSE183200. **K** The expression of NR1D1 in adenocarcinoma (Adeno) and NEPC subtypes from Beltran cohort. **L** Correlations between the expression of NR1D1 and LP drivers (SOX2, NR2F1/COUP-TF1 and PEG10) were performed in Beltran cohort.

Fig.S2-1

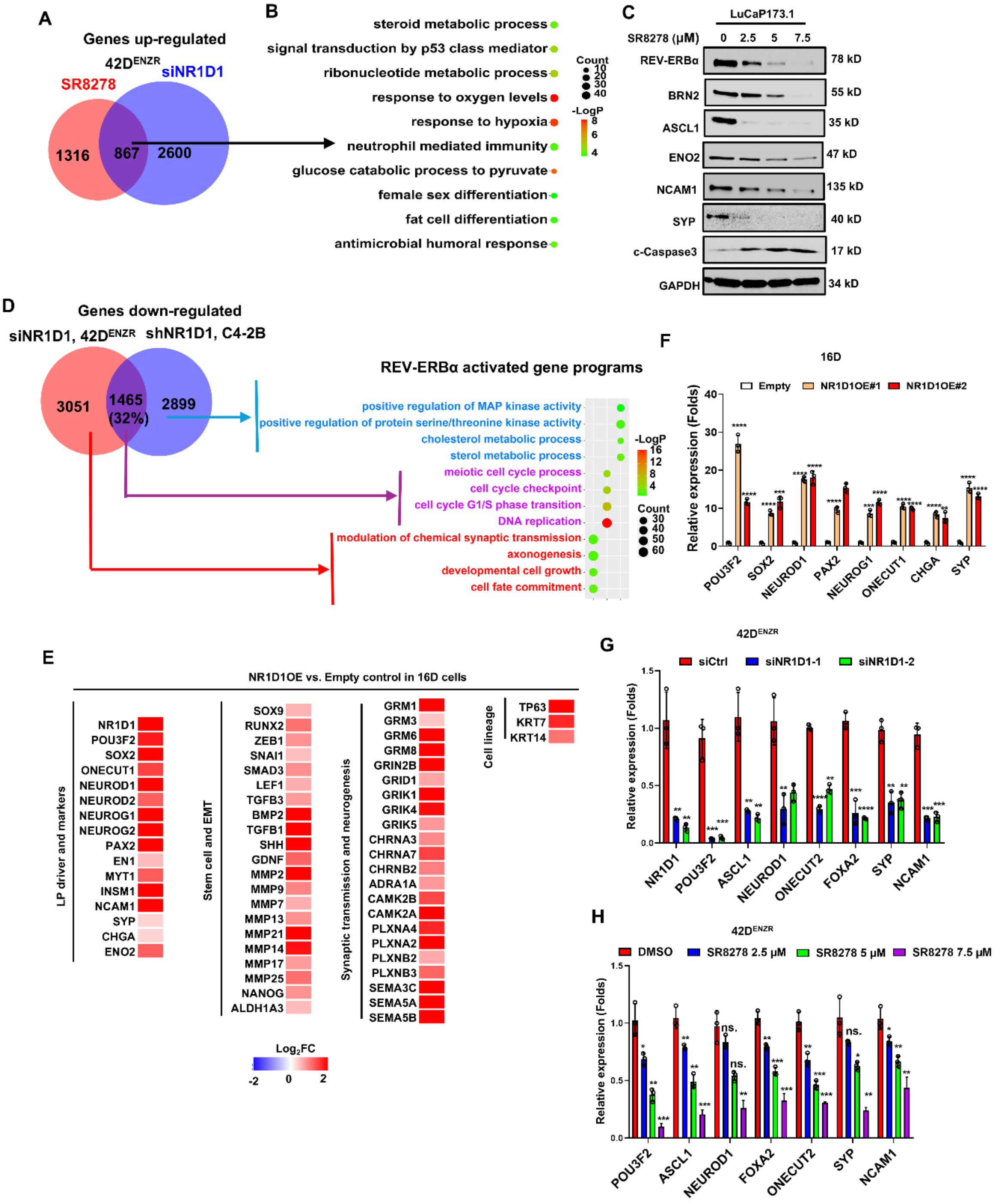
A
Genes up-regulated
42D^ENZR
SR8278
siNR1D1
1316
867
2600
B
steroid metabolic process
signal transduction by p53 class mediator
ribonucleotide metabolic process
response to oxygen levels
response to hypoxia
neutrophil mediated immunity
glucose catabolic process to pyruvate
female sex differentiation
fat cell differentiation
antimicrobial humoral response
Count
-LogP
C
LuCaP173.1
SR8278 (μM) 0 2.5 5 7.5
REV-ERBα 78 kD
BRN2 55 kD
ASCL1 35 kD
ENO2 47 kD
NCAM1 135 kD
SYP 40 kD
c-Caspase3 17 kD
GAPDH 34 kD
D
Genes down-regulated
siNR1D1, 42D^ENZR shNR1D1, C4-2B
3051
1465 (32%)
2899
REV-ERBα activated gene programs
positive regulation of MAP kinase activity
positive regulation of protein serine/threonine kinase activity
cholesterol metabolic process
sterol metabolic process
meiotic cell cycle process
cell cycle checkpoint
cell cycle G1/S phase transition
DNA replication
modulation of chemical synaptic transmission
axonogenesis
developmental cell growth
cell fate commitment
-LogP
Count
E
NR1D1OE vs. Empty control in 16D cells
LP driver and markers
NR1D1 POU3F2 SOX2 ONECUT1 NEUROD1 NEUROD2 NEUROG1 NEUROG2 PAX2 EN1 MYT1 INSM1 NCAM1 SYP CHGA ENO2
Stem cell and EMT
SOX9 RUNX2 ZEB1 SNAI1 SMAD3 LEF1 TGFB3 BMP2 TGFB1 SHH GDNF MMP2 MMP9 MMP7 MMP13 MMP21 MMP14 MMP17 MMP25 NANOG ALDH1A3
Synaptic transmission and neurogenesis
GRM1 GRM3 GRM6 GRM8 GRIN2B GRID1 GRIK1 GRIK4 GRIK5 CHRNA3 CHRNA7 CHRNB2 ADRA1A CAMK2B CAMK2A PLXNA4 PLXNA2 PLXNB2 PLXNB3 SEMA3C SEMA5A SEMA5B
Cell lineage
TP63 KRT7 KRT14
Log2FC
-2 0 2
F
16D
Empty NR1D1OE#1 NR1D1OE#2
Relative expression (Folds)
POU3F2 SOX2 NEUROD1 PAX2 NEUROG1 ONECUT1 CHGA SYP
G
42D^ENZR
siCtrl siNR1D1-1 siNR1D1-2
Relative expression (Folds)
NR1D1 POU3F2 ASCL1 NEUROD1 ONECUT2 FOXA2 SYP NCAM1
H
42D^ENZR
DMSO SR8278 2.5 μM SR8278 5 μM SR8278 7.5 μM
Relative expression (Folds)
POU3F2 ASCL1 NEUROD1 FOXA2 ONECUT2 SYP NCAM1

Fig.S2-2

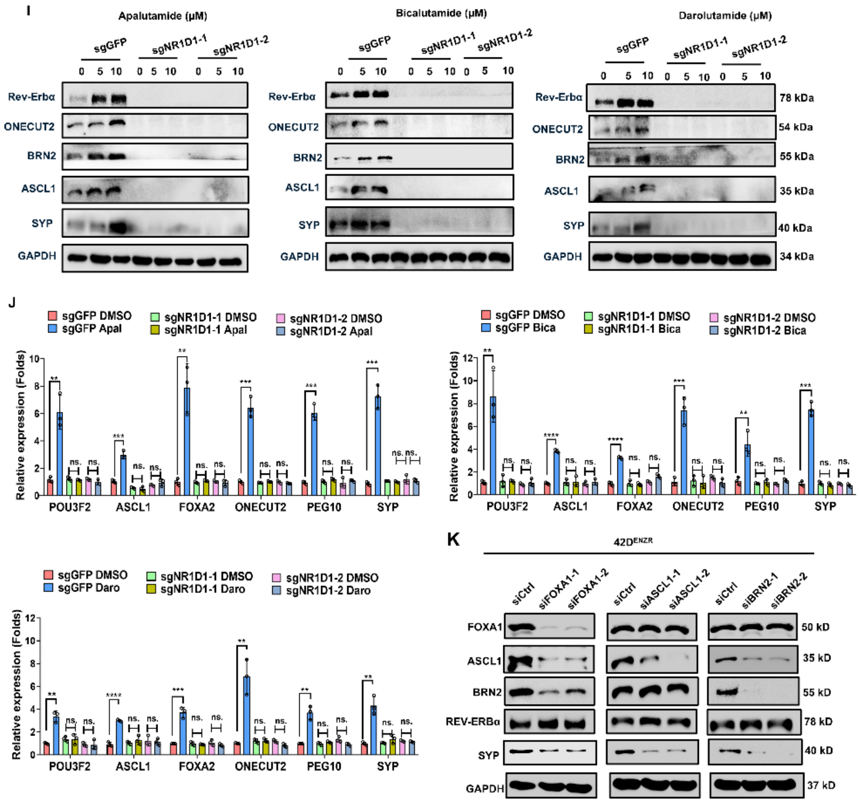

Fig.S2 REV-ERBα plays an essential role in ARSI induction of LP gene programs. A Venn diagram of the number of genes with expression significantly (> 1.5-fold) up-regulated in 42D^{ENZR} cells treated with siRNA against NR1D1/REV-ERBα and 7.5 μM antagonist SR8278 for 48 h. **B** Gene ontology (GO) analysis of the commonly upregulated genes by siRNA knockdown and antagonist treatment. Top 10 representative programs were shown. **C** Immunoblotting analysis of the expression of REV-ERBα and LP drivers and markers in LuCaP173.1 PDX derived cells treated with REV-ERBα antagonist with indicated concentrations of antagonist SR8278 for 48 h. **D** Left, Venn diagram of number of gene downregulated (> 1.5-fold) in adenocarcinoma C4-2B cells and t-NEPC 42D^{ENZR} cells by NR1D1 knockdown. Right, Gene ontology analysis of the unique and common programs affected by NR1D1 knockdown in C4-2B cells and 42-D^{ENZR} cells. **E** Heatmap of mRNA expression upregulated detected by RNA-seq in NR1D1 OE cells. **F-H** q-RT PCR analysis of the expression of REV-ERBα and LP drivers and markers in CRPC 16D cells with REV-ERBα OE (**F**), KD (**G**) and indicated concentrations of antagonist SR8278 (**H**) in 42D^{ENZR} for 48 h. **I-J** Immunoblotting (**I**) and q-RT PCR (**J**) analysis of the expression of REV-ERBα and LP drivers and markers in response to indicated concentrations of ARSI Apalutamide (Apal), Bicalutamide (Bica) and Darolutamide (Daro) treatments for 4 days in C4-2B cells with NR1D1/REV-ERBα KO. **K** Immunoblotting analysis of the expression of REV-ERBα, FOXA1 and LP drivers and marker SYP in 42D^{ENZR} cells treated with siRNAs against FOAX1, ASCL1 and POU3F2/BRN2 for 72 h.

Fig.S3-1

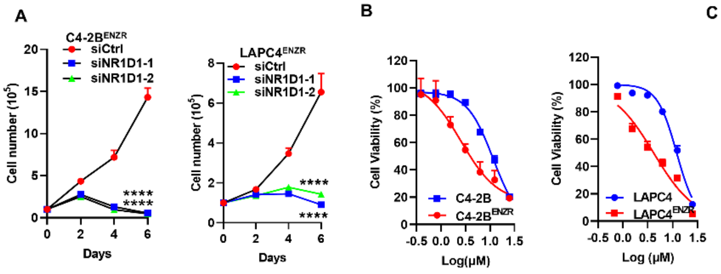

C

IC_{50} (μM)	Parental	ENZR
16D	10.12	5.49
C4-2B	11.63	2.57
LAPC4	12.09	4.16

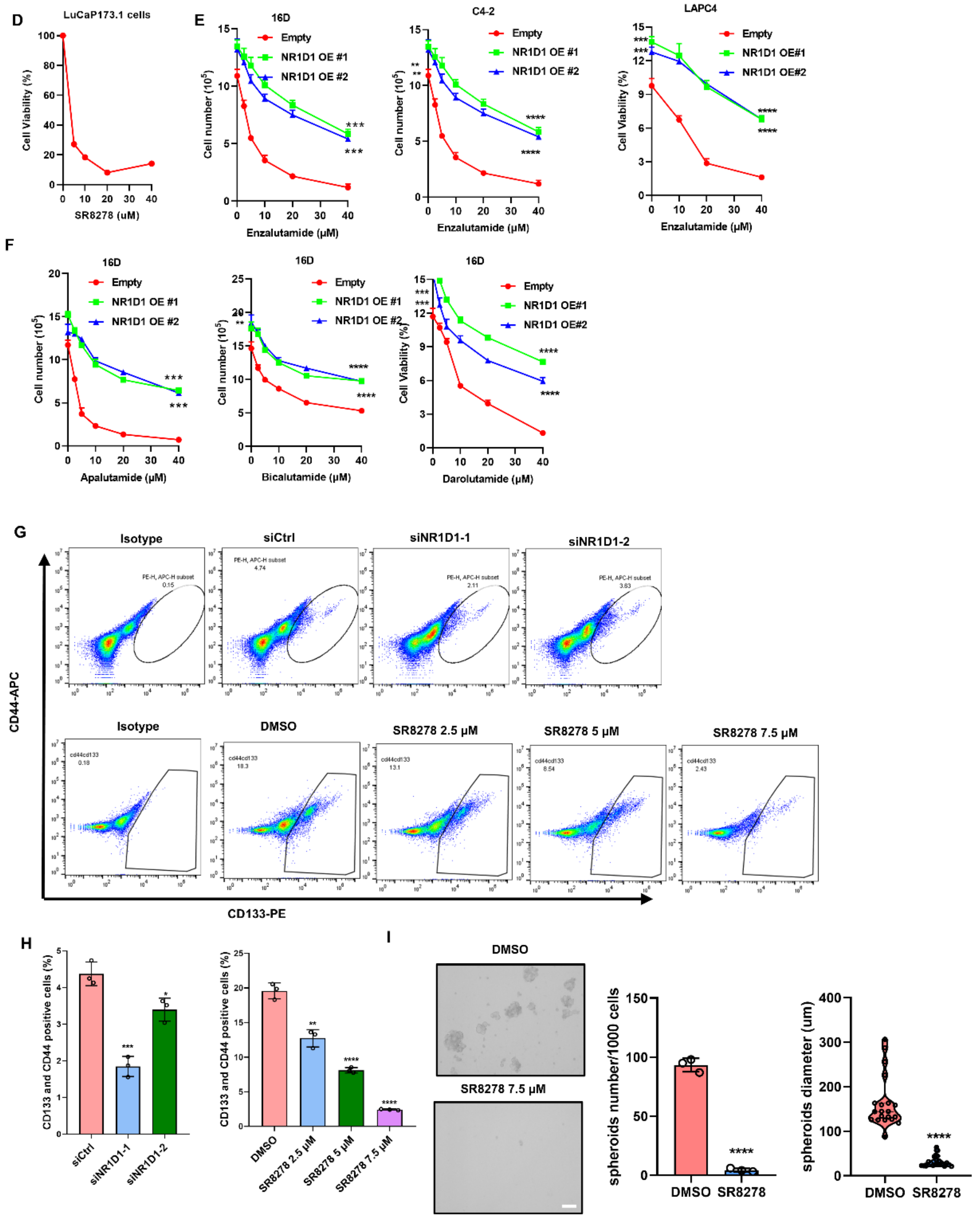

Fig.S3-2

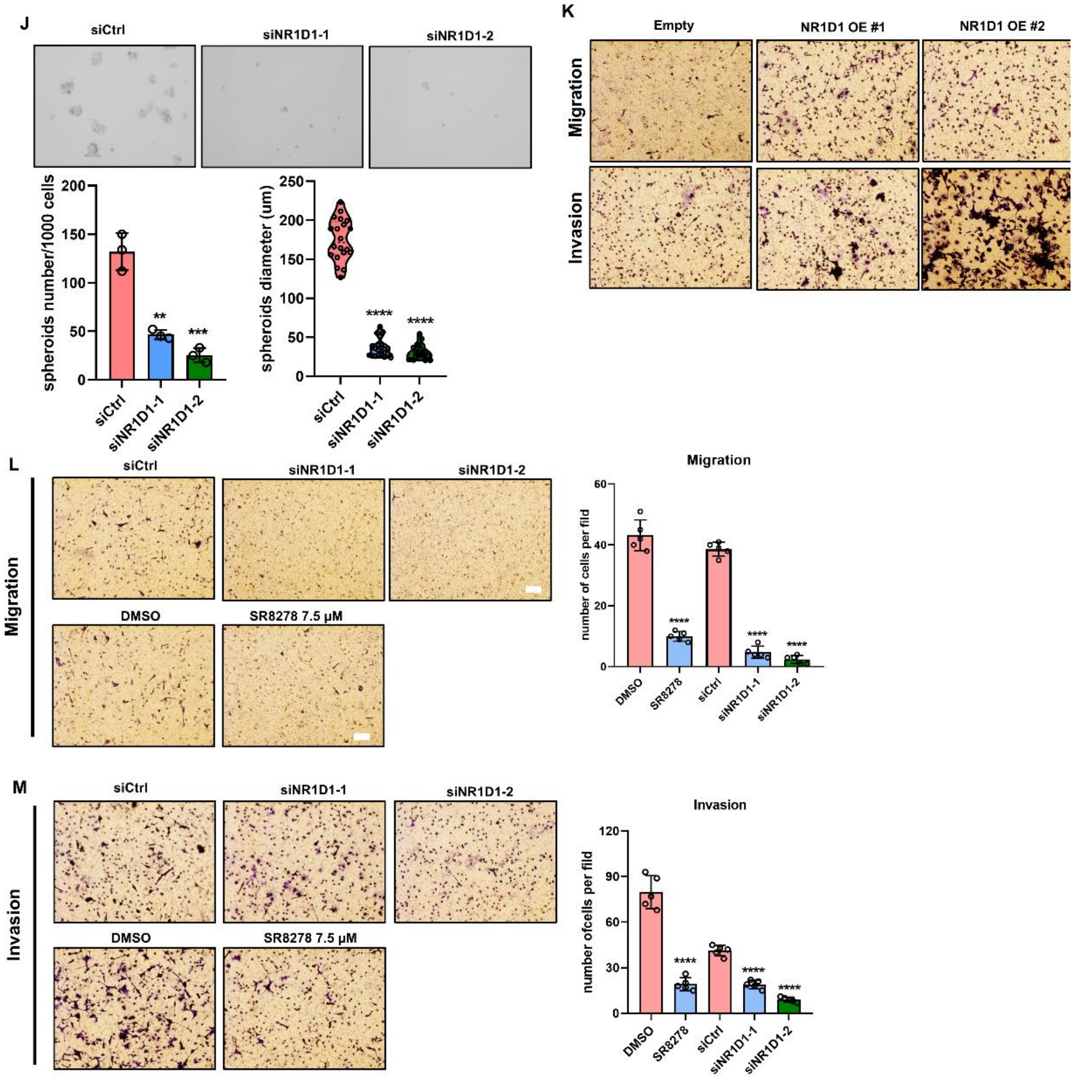
J
siCtrl
siNR1D1-1
siNR1D1-2
spheroids number/1000 cells
spheroids diameter (um)
K
Empty
NR1D1 OE #1
NR1D1 OE #2
Migration
Invasion
L
SR8278 7.5 μM
DMSO
number of cells per fild
M
number ofcells per fild
SR8278

Fig.S3-3

Fig.S3 Continued-2

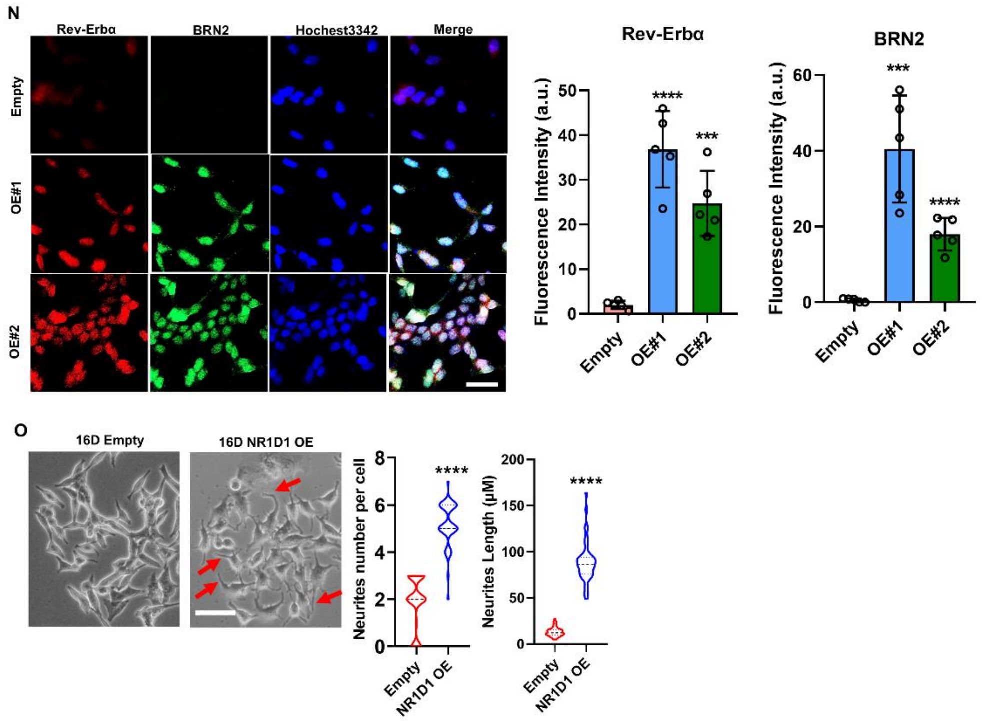

Fig.S3 Elevated REV-ERBα promotes ARSI resistance and aggressive cellular features that are associated with tumor LP. A Viable cell numbers were measured for ENZ-resistant C4-2B^{ENZR} and LAPC4^{ENZR} cells transfected with siRNAs against NR1D1 or control (siCtrl). **B** Cell-Titer Glo assays of cell viability of ENZ-resistant C4-2B^{ENZR} and LAPC4^{ENZR} and their parental C4-2B and LAPC4 cells treated with indicated concentrations of REV-ERBα antagonist SR8278 for 4 days. **C** Table of IC_{50} values of ENZ-resistant cells and their ENZ-sensitive parental cells calculated using Graph pad software as treated in **B**. 16D derived ENZ-resistant cells was named as 42D^{ENZR}. **D** Cell-Titer Glo assays of cell viability of de novo NEPC LuCaP173.1 cells isolated from fresh tumor treated with indicated concentrations of REV-ERBα antagonist SR8278 for 4 days. **E** Viable cell numbers were measured for C4-2 and LAPC4 cells with NR1D1 OE or vector control cells treated with indicated concentrations of ENZ for 4 days. **F** Viable cell numbers were measured for 16D cells with NR1D1 OE or vector control cells treated with indicated concentrations of ARSIs Apalutamide, Bicalutamide and Darolutamide for 4 days. **G-H** Representative flow cytometry plots of stem cell population (CD44^{pos} and CD133^{pos}) in 42D^{ENZR} cells treated siRNAs against NR1D1 or indicated concentrations of antagonist SR8278 for 72 h (**G**). Plot shows the quantification of stem cell populations in **G** (**H**). **I-J** Representative images of in vitro tumor sphere formation in 42D^{ENZR} cells treated with 7.5 μM antagonist SR8278 (**I**) and siRNAs against NR1D1/Rev-Erbα (**J**). Scale bars, 200 μm. Sphere number was counted and sphere diameter was measured using Image J software. **K-M** Migrated and invaded cells were stained and visualized by phase-contrast microscopy (10 x magnification). The number of migrated and invaded cells were counted. **N** Left, fluorescence microscope images of Rev-Erbα (red) and BRN2 (green) in 16D cells with NR1D1 OE. Right, the staining intensity was measured using Image J software. Bar = 50 μm. **O** Representative images of the neurite outgrowth marked with red arrows in 16D cells with NR1D1

OE or Empty control. Experimental data shown are mean ± SEM of 3 independent experiments performed in triplicate. Student’s t test. **i** $^{**}p < 0.01$, $^{***}p<0.001$, $^{****}p<0.0001$.

Fig.S4

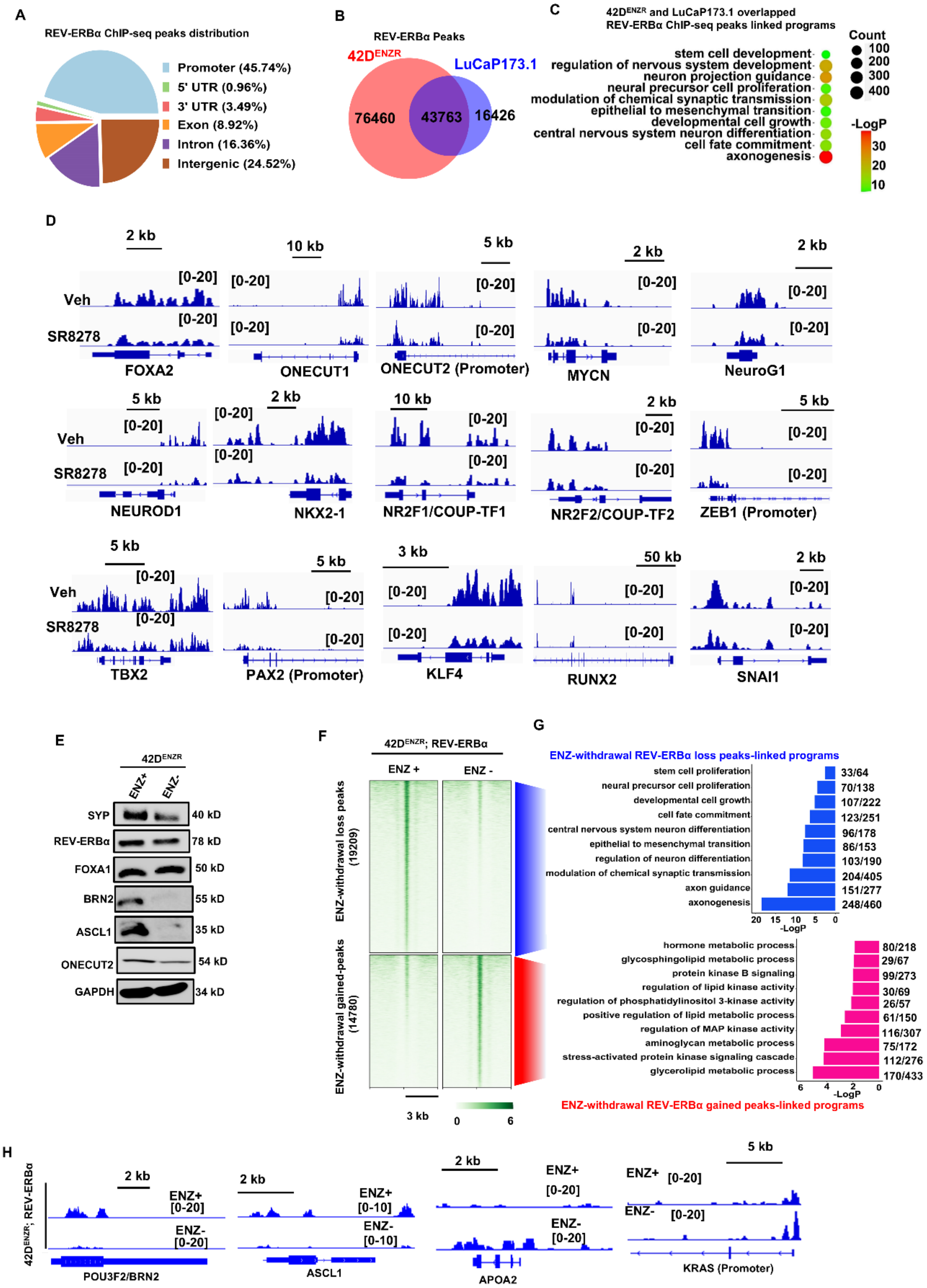

A
REV-ERBα ChIP-seq peaks distribution
Promoter (45.74%)
5' UTR (0.96%)
3' UTR (3.49%)
Exon (8.92%)
Intron (16.36%)
Intergenic (24.52%)
B
REV-ERBα Peaks
42DENZR
LuCaP173.1
76460
43763
16426
C
42DENZR and LuCaP173.1 overlapped
REV-ERBα ChIP-seq peaks linked programs
stem cell development
regulation of nervous system development
neuron projection guidance
neural precursor cell proliferation
modulation of chemical synaptic transmission
epithelial to mesenchymal transition
developmental cell growth
central nervous system neuron differentiation
cell fate commitment
axonogenesis
Count
100
200
300
400
-LogP
30
20
10
D
Veh
SR8278
[0-20]
FOXA2
ONECUT1
ONECUT2 (Promoter)
MYCN
NeuroG1
NEUROD1
NKX2-1
NR2F1/COUP-TF1
NR2F2/COUP-TF2
ZEB1 (Promoter)
TBX2
PAX2 (Promoter)
KLF4
RUNX2
SNAI1
E
42DENZR
ENZ+
ENZ-
SYP 40 kD
REV-ERBα 78 kD
FOXA1 50 kD
BRN2 55 kD
ASCL1 35 kD
ONECUT2 54 kD
GAPDH 34 kD
F
42DENZR; REV-ERBα
ENZ +
ENZ -
ENZ-withdrawal loss peaks (19209)
ENZ-withdrawal gained-peaks (14780)
3 kb
0
6
G
ENZ-withdrawal REV-ERBα loss peaks-linked programs
stem cell proliferation 33/64
neural precursor cell proliferation 70/138
developmental cell growth 107/222
cell fate commitment 123/251
central nervous system neuron differentiation 96/178
epithelial to mesenchymal transition 86/153
regulation of neuron differentiation 103/190
modulation of chemical synaptic transmission 204/405
axon guidance 151/277
axonogenesis 248/460
-LogP
hormone metabolic process 80/218
glycosphingolipid metabolic process 29/67
protein kinase B signaling 99/273
regulation of lipid kinase activity 30/69
regulation of phosphatidylinositol 3-kinase activity 26/57
positive regulation of lipid metabolic process 61/150
regulation of MAP kinase activity 116/307
aminoglycan metabolic process 75/172
stress-activated protein kinase signaling cascade 112/276
glycerolipid metabolic process 170/433
ENZ-withdrawal REV-ERBα gained peaks-linked programs
H
42DENZR; REV-ERBα
ENZ+
ENZ-
[0-20]
[0-10]
POU3F2/BRN2
ASCL1
APOA2
KRAS (Promoter)

Fig.S4 ARSI reprograms REV-ERBα to activate LP drivers and gene programs in a ARSI- dependent manner. A Genome-wide distribution of REV-ERBα ChIP-seq peaks in $42D^{ENZR}$ cells. **B** Venn diagram of numbers of REV-ERBα ChIP-seq peaks in $42D^{ENZR}$ cells and LuCaP173.1 tumors. **C** Gene ontology analysis of overlapping RevErbα ChIP-seq peaks-linked genes in **B**. **D** IGV snapshots of REV-ERBα chromatin occupancy at LP drivers in $42D^{ENZR}$ cells. **E** Immunoblotting analysis of the expression of REV-ERBα and LP drivers and markers in $42D^{ENZR}$ cells with or without ARSI ENZ for 7 days. **F** Heatmap indicating changes of REV-ERBα chromatin occupancy in response to ENZ withdrawal for 7 days in $42D^{ENZR}$ cells. **G** GO analysis of gene programs associated with gain or loss of REV-ERBα binding regions. Also shown are the number of REV-ERBα binding region associated genes and the total number of genes in each GO program. **H** IGV snapshots of REV-ERBα chromatin occupancy at LP drivers POU3F2/BRN2 and ASCL1, lipid metabolic process gene APOA2 and kinase signaling gene KRAS in the presence or absence of ENZ in $42D^{ENZR}$ cells.

Fig.S5-1

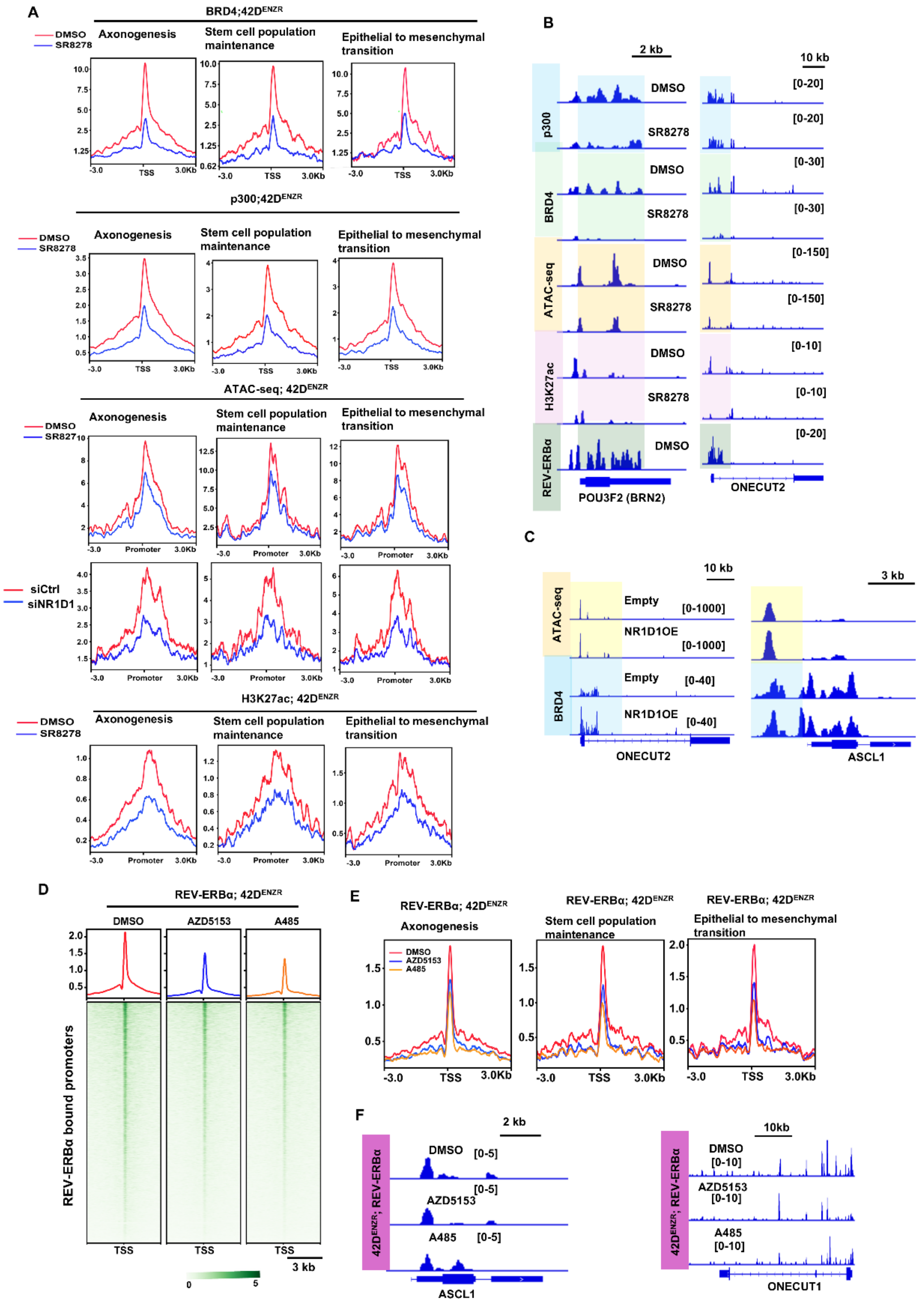

A
BRD4;42D ENZR
DMSO
SR8278
Axonogenesis
Stem cell population maintenance
Epithelial to mesenchymal transition
p300;42D ENZR
ATAC-seq; 42D ENZR
siCtrl
siNR1D1
H3K27ac; 42D ENZR
B
2 kb
10 kb
p300
BRD4
ATAC-seq
H3K27ac
REV-ERBα
POU3F2 (BRN2)
ONECUT2
C
Empty
NR1D1OE
ASCL1
D
REV-ERBα; 42D ENZR
AZD5153
A485
REV-ERBα bound promoters
3 kb
E
F
42D ENZR; REV-ERBα
ONECUT1

Fig.S5-2

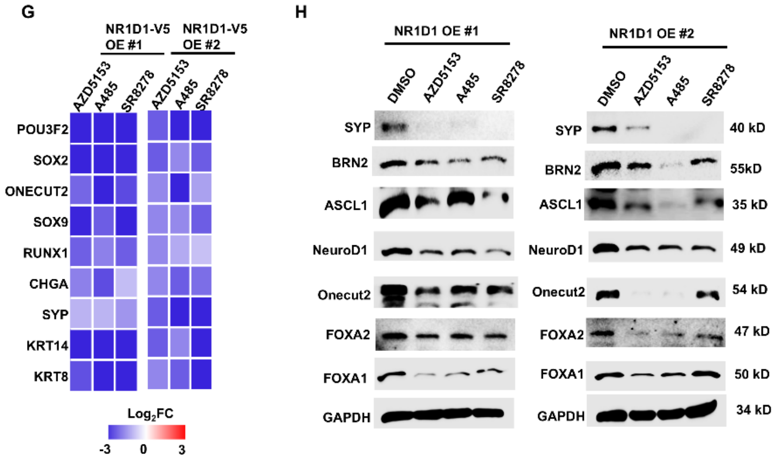

Fig.S5 REV-ERBα recruits BRD4 and p300 to remodel local chromatin accessibility and to activate the LP genes. A Signal profiles of chromatin occupancies of BRD4 and p300, chromatin accessibility and H3K27ac within +/- 3 kb around REV-ERBα bound promoters at LP programs in 42D^{ENZR} cells treated with 7.5 μM SR8278 or siRNA against REV-ERBα/NR1D1 for 24 h. **B** IGV snapshots of chromatin accessibility, BRD4 and FOXA1 occupancy at chromatin regions of LP drivers POU3F2/BRN2 and ONECUT2 as treated in **A**. **C** IGV snapshots of chromatin accessibility (upper panel) and BRD4 chromatin occupancy(lower panel) at LP drivers ONECUT2 and ASCL1 in Rev-Erbα/NR1D1 OE cells and Empty control cells. **D-E** Heatmaps (**D**) and signal profiles (**E**) of REV-ERBα binding peak intensity within +/- 3 kb around REV-ERBα bound promoters at LP programs in 42D^{ENZR} cells treated with 0.5 μM AZD5153 and 5 μM A485 for 24 h. **F** IGV snapshots of REV-ERBα occupancy at chromatin regions of LP drivers ASCL1 and ONECUT1 in 42D^{ENZR} cells treated with 0.5 μ M AZD5153 and 5 μM A485 for 24 h. **G** Heatmap of expression of LP genes in NR1D1 OE cells treated with 7.5 μM SR8278, 0.5 μM AZD5153 and 5 μM A485 for 48 h. **I** Immunoblotting of indicated proteins in REV-ERBα OE cells treated with 7.5 μM SR8278, 0.5 μM AZD5153 and 5 μM A485 for 48 h.

Fig.S6

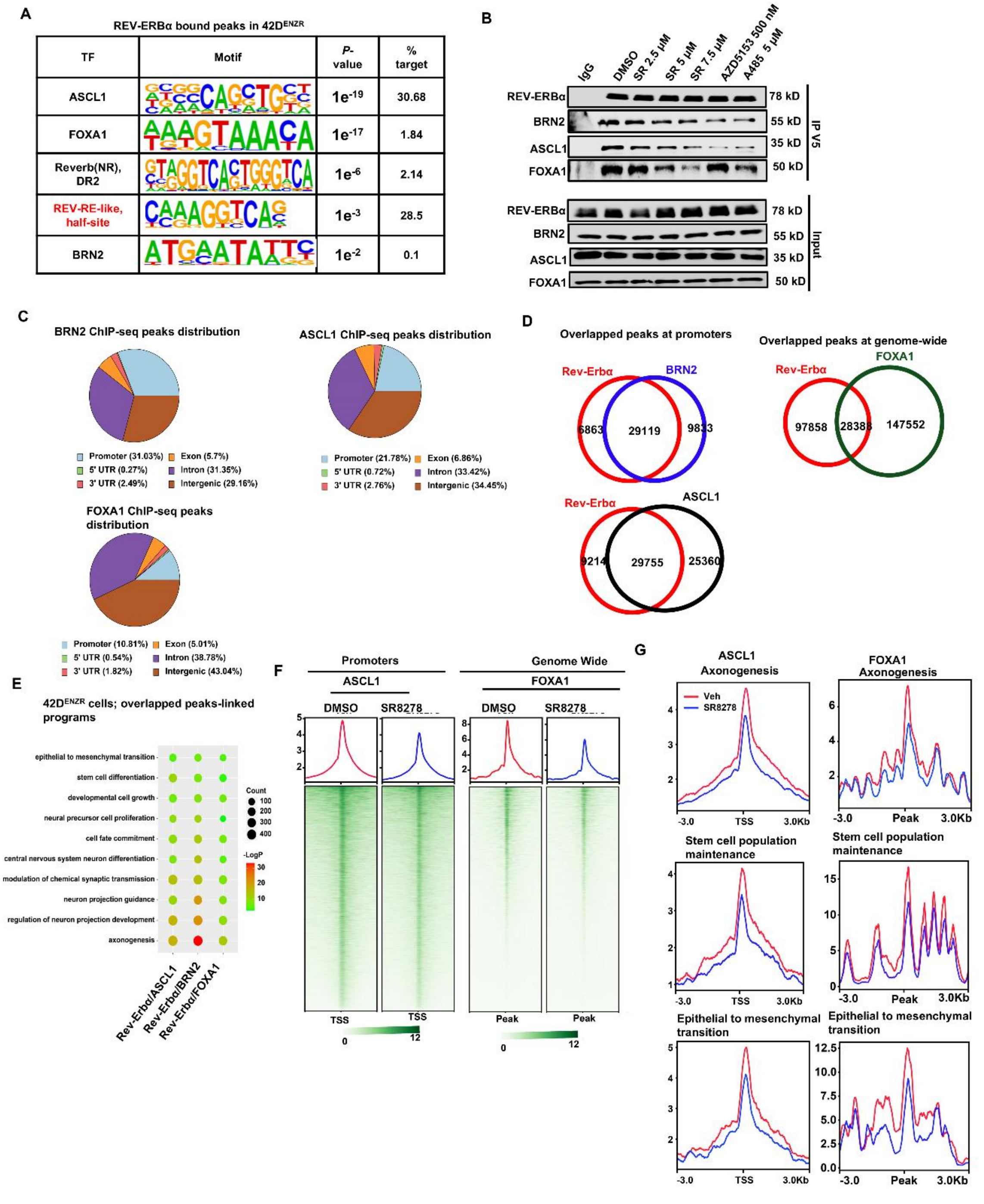
A
REV-ERBα bound peaks in 42D^ENZR
TF
Motif
P-value
% target
ASCL1
1e-19
30.68
FOXA1
1e-17
1.84
Reverb(NR), DR2
1e-6
2.14
REV-RE-like, half-site
1e-3
28.5
BRN2
1e-2
0.1
B
IgG
DMSO
SR 2.5 μM
SR 5 μM
SR 7.5 μM
AZD5153 500 nM
A485 5 μM
REV-ERBα
BRN2
ASCL1
FOXA1
78 kD
55 kD
35 kD
50 kD
IP V5
Input
C
BRN2 ChIP-seq peaks distribution
Promoter (31.03%)
Exon (5.7%)
5' UTR (0.27%)
Intron (31.35%)
3' UTR (2.49%)
Intergenic (29.16%)
ASCL1 ChIP-seq peaks distribution
Promoter (21.78%)
Exon (6.86%)
5' UTR (0.72%)
Intron (33.42%)
3' UTR (2.76%)
Intergenic (34.45%)
FOXA1 ChIP-seq peaks distribution
Promoter (10.81%)
Exon (5.01%)
5' UTR (0.54%)
Intron (38.78%)
3' UTR (1.82%)
Intergenic (43.04%)
D
Overlapped peaks at promoters
Rev-Erbα
BRN2
6863
29119
9833
Overlapped peaks at genome-wide
Rev-Erbα
FOXA1
97858
28388
147552
Rev-Erbα
ASCL1
9214
29755
25360
E
42D^ENZR cells; overlapped peaks-linked programs
epithelial to mesenchymal transition
stem cell differentiation
developmental cell growth
neural precursor cell proliferation
cell fate commitment
central nervous system neuron differentiation
modulation of chemical synaptic transmission
neuron projection guidance
regulation of neuron projection development
axonogenesis
Count
100
200
300
400
-LogP
30
20
10
Rev-Erbα/ASCL1
Rev-Erbα/BRN2
Rev-Erbα/FOXA1
F
Promoters
ASCL1
DMSO
SR8278
Genome Wide
FOXA1
TSS
Peak
0
12
G
ASCL1
Axonogenesis
Veh
SR8278
FOXA1
Axonogenesis
Stem cell population maintenance
Epithelial to mesenchymal transition
-3.0
TSS
3.0Kb
Peak

Fig.S6 REV-ERBα facilitates chromatin occupancy of known LP drivers BRN2, ASCL1 and FOXA1. **A** Motif analysis of REV-ERBα ChIP-seq peaks in $42D^{ENZR}$ cells. **B** Co-IP analysis of BRN2, ASCL1 and FOXA1 with REV-ERBα in $42D^{ENZR}$ cells expressing NR1D1-V5. Cells were treated with indicated doses of 7.5 μM SR8278, 0.5 μM AZD5153 and 5 μM A485 for 24 h. The nuclear extracts were used for co-IP with indicated antibodies. **C** Peak distribution analysis of BRN2, ASCL1 and FOXA1 ChIP-seq peaks in $42D^{ENZR}$ cells. **D** Venn diagram of number of RevErbα ChIP-seq peaks, BRN2 ChIP-seq peaks and ASCL1 ChIP-seq peaks at NR1D1 bound promoters and FOXA1 ChIP-seq peaks around NR1D1 genome wide peaks in $42D^{ENZR}$ cells. **E** Gene ontology analysis of overlapping peaks linked gene programs in **C**. **F-G** Heatmaps (**F**) and signal profiles (**G**) of ASCL1 and FOXA1 ChIP-seq peaks intensity within +/- 3 kb around the promoter and peak center at RevErbα ChIP-seq peak bound regions and LP programs in $42D^{ENZR}$ cells treated with 7.5 μM SR8278.

Fig.S7

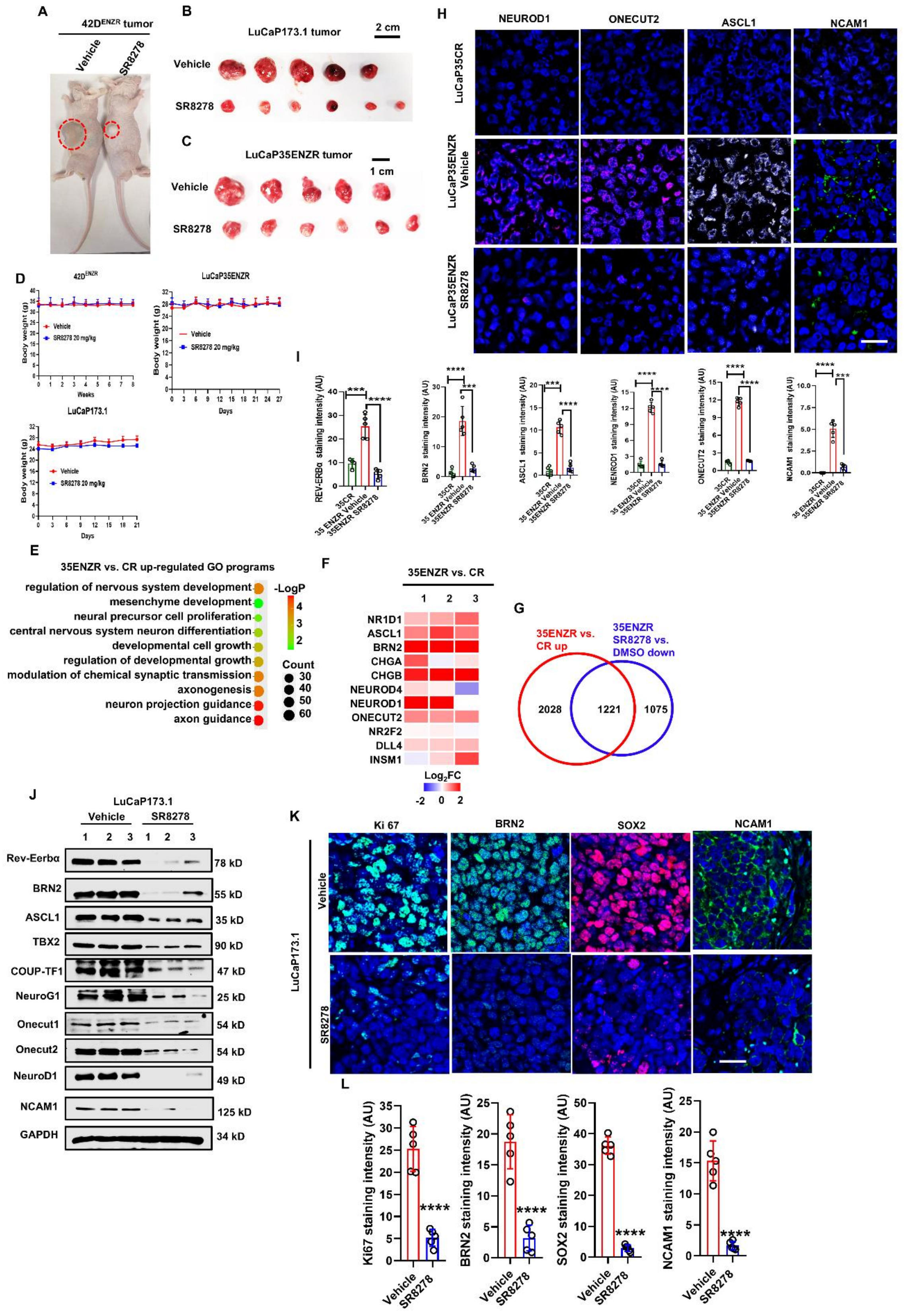
A
42D^ENZR tumor
Vehicle
SR8278
B
LuCaP173.1 tumor
2 cm
Vehicle
SR8278
C
LuCaP35ENZR tumor
1 cm
Vehicle
SR8278
D
42D^ENZR
LuCaP35ENZR
LuCaP173.1
Body weight (g)
Vehicle
SR8278 20 mg/kg
Weeks
Days
E
35ENZR vs. CR up-regulated GO programs
regulation of nervous system development
mesenchyme development
neural precursor cell proliferation
central nervous system neuron differentiation
developmental cell growth
regulation of developmental growth
modulation of chemical synaptic transmission
axonogenesis
neuron projection guidance
axon guidance
-LogP
Count
F
35ENZR vs. CR
NR1D1
ASCL1
BRN2
CHGA
CHGB
NEUROD4
NEUROD1
ONECUT2
NR2F2
DLL4
INSM1
Log2FC
G
35ENZR vs. CR up
35ENZR SR8278 vs. DMSO down
2028
1221
1075
H
NEUROD1
ONECUT2
ASCL1
NCAM1
LuCaP35CR
LuCaP35ENZR Vehicle
LuCaP35ENZR SR8278
I
REV-ERBα staining intensity (AU)
BRN2 staining intensity (AU)
ASCL1 staining intensity (AU)
NEUROD1 staining intensity (AU)
ONECUT2 staining intensity (AU)
NCAM1 staining intensity (AU)
35CR
35 ENZR Vehicle
35ENZR SR8278
J
LuCaP173.1
Vehicle
SR8278
Rev-Eerbα 78 kD
BRN2 55 kD
ASCL1 35 kD
TBX2 90 kD
COUP-TF1 47 kD
NeuroG1 25 kD
Onecut1 54 kD
Onecut2 54 kD
NeuroD1 49 kD
NCAM1 125 kD
GAPDH 34 kD
K
Ki 67
BRN2
SOX2
NCAM1
LuCaP173.1
Vehicle
SR8278
L
Ki67 staining intensity (AU)
BRN2 staining intensity (AU)
SOX2 staining intensity (AU)
NCAM1 staining intensity (AU)
Vehicle
SR8278
****

Fig.S7 Pharmacological inhibition of REV-ERBα potently suppresses tumor growth and LP gene programs in vivo. A-C Representative tumor images from mice bearing 42D^{ENZR} (**A**), LuCaP35ENZR (**B**) and LuCaP173.1 tumors (**C**) were treated, i.p., 5 times per week, with vehicle or 20 mg/kg SR8278 for indicated days. **D** Body weight measurement of mice treated with SR8278 in (**A-C**). **E** Representative programs significantly enriched of genes upregulated (> 1.5-fold) in ARSI-resistant LuCaP35ENZR tumors versus ARSI-sensitive LuCaP35CR tumors. **F** Heatmap of mRNA expression up-regulated in LuCaP35ENZR tumors detected by RNA-seq. **G** Venn diagram analysis of number of genes upregulated in LuCaP35ENZR tumors and downregulated by SR8278 treatment in vivo. **H-I** Representative images of immunofluorescence staining of LP drvers NEUROD1 ONECUT2 and ASCL1 and neuroendocrine marker NCAM1 in LuCaP35CR, LuCaP35ENZR tumors (**H**). Staining intensity of these genes were measured by image J using at least 5 random images (**I**). Bar = 100 μm. Student's t test. **p < 0.01, ***p<0.001, ****p<0.0001. **J I**ntratumoral levels of proteins involved in neuronal differentiation programs in LuCaP173.1 tumors treated with 20 mg/kg SR8278 (i,.p.) were detected by immunoblotting. **K-L** Representative images of immunofluorescence staining of Ki67, BRN2, SOX2 and NCAM1 (**K**). Bar = 100 μm. Staining intensity of these genes were measured by image J using at least 5 random images (**L**). Student's t test. **p < 0.01, ***p<0.001, ****p<0.0001

Table S1. qRT-PCR primers of genes.

Gene symbol	Forword primer 5'-3'	Reverse primer 5'-3'
NR1D1/ REV-ERBα	ATCGTCCGCATCAATCGCAA	CTGCTTCTCTCGTTTGGGGAT
POU3F2/BRN2	ACACTGACCGATCTCCACGCAGTA	GAGGGTGTGGGACCCTAAATATGAC
ASCL1	CGCGGCCAACAAGAAGATG	CGACGAGTAGGATGAGACCG
FOXA2	GGAGCAGCTACTATGCAGAGC	CGTGTTCATGCCGTTCATCC
PEG10	GAGCACCAGGGATTTCTCAGT	GGTAGTTGTGCATCAGGTAGTG
DLL3	CGTCCGTAGATTGGAATCGCC	TCCCGAGCGTAGATGGAAGG
ONECUT2	CAAACGCCCGTCAAAGGAGAT	GCTCAGATCGTCTTGCCACTT
NEUROD1	GGATGACGATCAAAAGCCCAA	GCGTCTTAGAATAGCAAGGCA
NR2F2/COUP-TF2	TCATGGGTATCGAGAACATTTGC	TTCAACACAAACAGCTCGCTC
TBX2	GCTGACGATTGCCGCTATAAG	GGCTGTCTGGGTGGATGTA
SYP	TCAGTTCCGGGTGGTCAAG	AAGACCCATTGCAGCACCTT
AR	ACATCAAGGAACTCGATCGTATCATTGC	TTG GGC ACT TGC ACA GAG AT
KLK3/PSA	GACCAAGTTCATGCTGTGTGC	CCACTCACCTTTCCCCTCAAG
SOX2	GCCGAGTGGAAACTTTTGTCG	GGCAGCGTGTACTTATCCTTCT
SOX9	AGCGAACGCACATCAAGAC	CTGTAGGCGATCTGTTGGGG
RUNX1	CTGCCCATCGCTTTCAAGGT	GCCGAGTAGTTTTCATCATTGCC
CHGA	TAAAGGGGATACCGAGGTGATG	TCGGAGTGTCTCAAAACATTCC
KRT14	GAAGAACCGCAAGGATGCTGAG	TGCAGCTCGATCTCCAGGTTCT
KRT8	TCCTCAGGCAGCTATATGAAGAG	GGTTGGCAATATCCTCGTACTGT
GAPDH	CGACCTGACCTGCCGTCTAGAA	GGTGTCGCTGGTGAAGTCGAGAG

Table S2. Antibodies information used for immunoblotting and immunofluorescence (IF) staining.

Antibody	Vendor	Catalog number	Dilution
REV-ERBα	Cell signaling technology	13418	WB,1:1000; IF, 1:200
FOXA1	abcam	23738	WB, 1:1000
POU3F2/BRN2	Cell signaling technology	12137	WB,1:1000; IF, 1:200
ASCL1	abcam	ab211327	WB,1:1000; IF, 1:200
GAPDH	Cell signaling technology	2118L	WB,1:1000
SYP	Cell signaling technology	36406S	WB,1:1000; IF, 1:200
PSA/KLK3	Santa Cruz	Sc-7316	WB,1:500
FOXA2	Cell signaling technology	8186S	WB,1:1000
NR2F1/COUP-TF1	Cell signaling technology	6364S	WB, 1:1000
ONECUT2	Proteintech	2196-1-AP	WB, 1:1000; IF, 1:200
NEUROG1	Proteintech	66642-1-Ig	WB, 1:1000
PAX6	Biolegends	901301	WB, 1:500
ENO2	Cell signaling technology	65162S	WB, 1:1000
NCAM1	Cell signaling technology	99746S	WB,1:1000; IF,1:200
c-Caspase3	Cell signaling technology	9664S	WB, 1:1000
NEUROD1	Cell signaling technology	62953S	WB, 1:1000; IF, 1:200
TBX2	Santa Cruz	sc-514291	WB, 1:500
ONECUT1	Proteintech	25137-1-AP	WB, 1:1000
CHGA	abcam	ab283265	WB, 1:1000
SOX2	abcam	ab92494	WB,1:1000; IF,1:200
MYCN	Cell signaling technology	51705S	WB, 1:1000
Ki67	abcam	ab15580	IF, 1:200

Table S3. AR status across models.

Cell line/model	AR status	Androgen/ARSI sensitivity	Notes
**LNCaP**	AR-positive (mutated AR)	Androgen-sensitive; ARSI-sensitive	Androgen-sensitive and AR-dependent (4).
**16D**	AR-positive (mutated AR)	Androgen-resistant; ARSI-sensitive	Derived from LNCaP xenograft that relapsed after castration. Androgen-independent, but still AR-dependent (4).
**42D^{ENZR}**	AR-positive (mutated AR)	Androgen-resistant; ARSI-resistant	Derived from 16D xenograft that relapsed after ARSI enzalutamide treatment in vivo. Androgen-independent, and resistant to ARSI enzalutamide (4).
**C4-2B**	AR-positive (mutated AR)	Androgen-resistant; ARSI-sensitive	Derived from LNCaP xenograft that relapsed after castration. Androgen-independent, but still AR-dependent. AR activity is higher and more stable in C4-2B cells than parental LNCaP cells. AR silencing inhibits proliferation (5).
**LAPC4**	AR-positive (wild-type AR)	Androgen-sensitive; ARSI-sensitive	Androgen-sensitive and androgen-dependent. AR silencing inhibits proliferation and tumor formation in xenografts (6).
**LuCaP35CR**	AR-positive (patient-derived xenograft model) (wild-type AR)	Androgen-resistant; ARSI-sensitive	Patient-derived xenograft (PDX) models often maintain the AR amplification status found in the original tumors. CR denotes castration-resistant, implying a focus on AR-dependent resistance mechanisms (7).
**LuCaP35ENZR**	AR-positive (patient-derived xenograft model) (wild-type AR)	Androgen-resistant; ARSI-resistant	Derived from LuCaP35CR xenograft that relapsed after ARSI enzalutamide treatment in vivo. Androgen-independent, and resistant to ARSI enzalutamide (7).
**LuCaP173.1**	AR-negative (patient-derived xenograft model)	Androgen-resistant; ARSI-resistant	Patient-derived xenograft (PDX) originates from NEPC that arises de novo. LuCaP 173.1 lacks expression of the androgen receptor (AR) and exhibits the characteristics of neuroendocrine cells, including positive staining for markers like Synaptophysin (SYP) (8).

DatasetS1. RNA-seq analysis of 42D^{ENZR} cells treated with NR1D1/ REV-ERBα antagonist SR8278, siRNAs against NR1D1 and NR1D1/ REV-ERBα OE in 16D CRPC cells.

DataS1A: 42DENZR cells treated with 7.5 uM SR8278 for 72h (RNA-seq, FPKM);

DataS1B: 42DENZR cells treated with siRNA against NR1D1/REV-ERBα for 72h (RNA-seq, FPKM);

DataS1C: NR1D1 KD and SR8278 commonly down-regulated programs in 42DENZR;

DataS1D: NR1D1/REV-ERBα OE in 16D CRPC cells (RNA-seq, FPKM);

DataS1E: NR1D1/REV-ERBα OE upregulated programs in 16D CRPC cells.

DatasetS2. Peak analysis of REV-ERBα, BRD4 and p300 ChIP-seq in 42D^{ENZR} cells treated with NR1D1/ REV-ERBα antagonist SR8278 or ARSI ENZ withdrawal.

DatasetS2A: REV-ERBα directly activated genes in 42D^{ENZR} cells;

DatasetS2B: REV-ERBα directly activated gene programs in 42DENZR cells;

DatasetS2C: REV-ERBα ChIP-seq overlapped peaks in 42D^{ENZR} and LuCaP173.1;

DatasetS2D: Homer motif of REV-ERBα ChIP-seq peaks in 42D^{ENZR} cells;

DatasetS2E: REV-ERBα ChIP-seq overlapped peaks-linked gene programs in 42D^{ENZR} and LuCaP173.1;

DatasetS2F: REV-ERBα/BRD4/p300 co-bound ChIP-seq peaks in 42D^{ENZR} cells;

DatasetS2G: REV-ERBα/BRD4/p300 co-bound promoters ChIP-seq peaks-linked gene programs in 42D^{ENZR};

DatasetS2H: REV-ERBα ChIP-seq lost peaks-linked programs after ENZ withdrawal;

DatasetS2I: REV-ERBα ChIP-seq gained peaks-linked programs after ENZ withdrawal.

DatasetS3. RNA-seq analysis of LuCaPENZR PDX tumors and LuCaP173.1 tumors treated with NR1D1/ REV-ERBα antagonist SR8278.

DatasetS3A: RNA-seq for LuCaP35CR parental tumor and LuCaP35ENZR tumors treated with 20 mg/kg SR8278 (i.p.);

DatasetS3B: Programs upregulated in LuCaP35ENZR vs. LuCaP35CR tumors;

DatasetS3C: Programs downregulated by SR8278 treatment in LuCaP35ENZR tumors;

DatasetS3D: RNA-seq for LuCaP173.1 tumors treated with 20 mg/kg SR8278 (i.p.);

DatasetS3E: Programs downregulated by SR8278 treatment in LuCaP173.1 tumors.

SI References

1. Y. Yang *et al.*, Functional inversion of circadian regulator REV-ERBalpha leads to tumorigenic gene reprogramming. *Proc Natl Acad Sci U S A* **121**, e2411321121 (2024).
2. M. R. Corces *et al.*, An improved ATAC-seq protocol reduces background and enables interrogation of frozen tissues. *Nat Methods* **14**, 959-962 (2017).
3. X. Zhang *et al.*, Effective therapeutic targeting of tumor lineage plasticity in neuroendocrine prostate cancer by BRD4 inhibitors. *Acta Pharm Sin B* **15**, 1415-1429 (2025).
4. J. L. Bishop *et al.*, The Master Neural Transcription Factor BRN2 Is an Androgen Receptor-Suppressed Driver of Neuroendocrine Differentiation in Prostate Cancer. *Cancer Discov* **7**, 54-71 (2017).
5. G. N. Thalmann *et al.*, Androgen-independent cancer progression and bone metastasis in the LNCaP model of human prostate cancer. *Cancer Res* **54**, 2577-2581 (1994).

6. K. A. Klein *et al.*, Progression of metastatic human prostate cancer to androgen independence in immunodeficient SCID mice. *Nat Med* **3**, 402-408 (1997).
7. H. M. Lam *et al.*, Durable Response of Enzalutamide-resistant Prostate Cancer to Supraphysiological Testosterone Is Associated with a Multifaceted Growth Suppression and Impaired DNA Damage Response Transcriptomic Program in Patient-derived Xenografts. *Eur Urol* **77**, 144-155 (2020).
8. M. P. Labrecque *et al.*, Molecular profiling stratifies diverse phenotypes of treatment-refractory metastatic castration-resistant prostate cancer. *J Clin Invest* **129**, 4492-4505 (2019).
